# Novel linear motif filtering protocol reveals the role of the LC8 dynein light chain in the Hippo pathway

**DOI:** 10.1371/journal.pcbi.1005885

**Published:** 2017-12-14

**Authors:** Gábor Erdős, Tamás Szaniszló, Mátyás Pajkos, Borbála Hajdu-Soltész, Bence Kiss, Gábor Pál, László Nyitray, Zsuzsanna Dosztányi

**Affiliations:** 1 MTA-ELTE Lendület Bioinformatics Research Group, Department of Biochemistry, Eötvös Loránd University, Budapest, Hungary; 2 Department of Biochemistry, Eötvös Loránd University, Budapest, Hungary; National Institutes of Health, UNITED STATES

## Abstract

Protein-protein interactions (PPIs) formed between short linear motifs and globular domains play important roles in many regulatory and signaling processes but are highly underrepresented in current protein-protein interaction databases. These types of interactions are usually characterized by a specific binding motif that captures the key amino acids shared among the interaction partners. However, the computational proteome-level identification of interaction partners based on the known motif is hindered by the huge number of randomly occurring matches from which biologically relevant motif hits need to be extracted. In this work, we established a novel bioinformatic filtering protocol to efficiently explore interaction network of a hub protein. We introduced a novel measure that enabled the optimization of the elements and parameter settings of the pipeline which was built from multiple sequence-based prediction methods. In addition, data collected from PPI databases and evolutionary analyses were also incorporated to further increase the biological relevance of the identified motif hits. The approach was applied to the dynein light chain LC8, a ubiquitous eukaryotic hub protein that has been suggested to be involved in motor-related functions as well as promoting the dimerization of various proteins by recognizing linear motifs in its partners. From the list of putative binding motifs collected by our protocol, several novel peptides were experimentally verified to bind LC8. Altogether 71 potential new motif instances were identified. The expanded list of LC8 binding partners revealed the evolutionary plasticity of binding partners despite the highly conserved binding interface. In addition, it also highlighted a novel, conserved function of LC8 in the upstream regulation of the Hippo signaling pathway. Beyond the LC8 system, our work also provides general guidelines that can be applied to explore the interaction network of other linear motif binding proteins or protein domains.

## Author summary

Fine-tuning of many cellular processes relies on weak, transient protein-protein interactions. Such interactions often involve compact functional modules, called short linear motifs (SLiMs) that can bind to specific globular domains. SLiM-mediated interactions can carry out diverse molecular functions by targeting proteins to specific cellular locations, regulating the activity and binding preferences of proteins, or aiding the assembly of macromolecular complexes. The key to the function of SLiMs is their small size and highly flexible nature. At the same time, these properties make their experimental identification challenging. Consequently, only a small portion of SLiM-mediated interactions is currently known. This underlies the importance of novel computational methods that can reliably identify candidate sites involved in binding to linear motif binding domains. Here we present a novel bioinformatic approach that efficiently predicts new binding partners for SLiM-binding domains. We applied this method to the dynein light chain LC8, a protein that was already known to bind many partners in a wide range of organisms. With this method, we not only significantly expanded the interaction network of LC8, but also identified a novel function of LC8 in a highly important pathway controlling organ size in animals.

## Introduction

A large number of protein-protein interactions (PPIs) are mediated by short linear motifs (SLiMs) that are recognized by specific globular domains [1]. SLiM-mediated interactions are involved in a wide range of biological functions and can regulate the formation of transient protein complexes, orchestrate subcellular localization, modulate post-translational modification state, and determine the fate of proteins [1]. Such interactions emerged as key mediators of complex regulatory processes in higher eukaryotic cells and their aberrant functioning can contribute to various diseases as well [2]. The key to the essential nature of SLiMs in biological systems lies in their specific properties. SLiMs correspond to a stretch of approximately 3–10 residues that generally reside within intrinsically disordered regions (IDRs). As a result, they usually form transient, weak interactions with micromolar binding affinity [3]. Due to these specific properties, the identification of linear motif sites is challenging both experimentally and computationally [4]. Currently, the most comprehensive collection, the Eukaryotic Linear Motif database (ELM) holds only 200–300 motif patterns with a few thousands of experimentally verified instances [5]. This number pales in comparison to the expected number of linear motif mediated interactions in the human proteome, estimated to number at least several hundred thousand [6]. Linear binding peptides have been systematically analyzed only for a few specific interaction domains, such as SH2, PTB, 14-3-3, PDZ and SH3 domains [7,8]. However, for most motif binding domains, the interaction network is largely incomplete.

The identification of linear motif mediated interactions is usually divided into two phases. The first phase is the characterization of the common consensus sequence motif that is shared among the diverse set of binding partners of a common domain, i.e. defining the motif class [9,10]. The core motif that mediates interactions with a given domain is usually represented by a sequence pattern or a position specific scoring matrix (PSSM). In the second phase, the core motif is used to identify additional candidate binding sites in the proteome, i.e. finding novel motif instances. However, as the information content of consensus motifs is usually low, predicted motif matches are overwhelmingly dominated by false positive matches that occur purely by chance [3,11]. Therefore, this phase involves additional filtering steps to remove matches that are unlikely to be functional and to prioritize motif hits for further experimental characterization. Various computational tools such as ELM, QuasiMotifFinder, MiniMotifMiner, SLiMSearch, ScanProsite or DOReMi [5,12–16] have been developed to overcome this problem. These tools scan a defined set of proteins with a single consensus motif and utilize various discriminatory attributes to prioritize motif hits, including structural context, protein disorder, functional ontology, evidence for PPIs and shared cellular localization. Evolutionary conservation can highlight functionally relevant positions in proteins and have been used for globular domains to identify conserved motif-like patterns that are indicative of the function of a protein [12]. However, linear motif sites generally reside within IDRs that are generally less conserved [17]. Within these regions, SLiMs often show a specific pattern of evolutionary conservation that are characterized by a higher relative conservation of the key motif residues compared to their flanking regions [18,19], and this information can be used to highlight true binding motifs. Using various sequence attributes, specific filtering pipelines were constructed to identify novel motif instances for several domains [20–24]. However, the strength and optimality of the filtering steps have never been systematically tested. In order to build optimal filtering protocols, a more systematic approach is needed that can take into account the specific trade-off between reducing false positive hits while capturing biologically relevant motif matches, which is likely to be specific to individual binding domains.

In this study, we focused on LC8 dynein light chain and its binding partners as a case study, and explored how the interaction network of a specific linear motif binding protein can be expanded in an optimal way. LC8 is a remarkably conserved eukaryotic hub protein [22]. Although LC8 was originally suggested to function as a cargo adaptor for the dynein motor complex, its extensive interaction network suggests a more general role, independent of dynein [22,25]. Recently, the prevailing view has become that LC8 functions as a dimerization or oligomerization engine for various proteins [25,26]. Known interaction partners link LC8 to processes such as nuclear transport, tumor suppression, viral replication, DNA damage repair, apoptosis, mitosis and signaling [22,25]. In contrast to their functional heterogeneity, LC8 binding partners generally share a common binding mechanism [22,27]. The known structures of complexes between LC8 and various bound partners show that the binding groove is formed at the dimerization interface of the homodimeric LC8, favoring binding partners that are also dimerized [28,29], often promoted by coiled coil (CC) regions [20,30]. The recognition SLiMs are generally located within intrinsically disordered regions [25] and undergo a disorder-to-order transition upon complex formation. In their bound form, these segments adopt highly similar conformations that augment the central beta sheet of LC8 on each side of the dimer [27]. Many of the binding segments contain a Thr-Gln-Thr (TQT) motif with additional positions showing larger variations. Apart from the canonical TQT motif, there are also non-canonical binding motif instances, in which the central Gln is replaced by Met or Asn [22]. An even more unusual Thr-Ser-Pro (TSP) binding motif mediates the interaction between Pak1 and LC8. Altogether, more than 50 LC8 binding motif instances were collected from various eukaryotic species [22]. To reveal the optimal binding motif, a phage display study was also carried out, identifying further LC8 binding motif instances [20]. The suggested general role for LC8 raises the possibility of many additional binding partners in the human proteome.

In this work we expanded the interaction network of dynein light chain LC8 using a combination of computational and experimental methods. We introduced a novel measure based on information gain that enabled us to build an optimal bioinformatic pipeline by combining various attributes predicted from the amino acid sequence. We also incorporated known binding partners from PPI databases and exploited the specific evolutionary conservation of binding motifs to increase the likelihood that the motif hit is biologically relevant. The resulting procedure enabled us to drastically reduce false positive predictions among putative novel linear motif instances and to expand the interaction network of LC8 with high-confidence predictions. We experimentally verified the binding of several novel motif instances to LC8 using surface plasmon resonance (SPR) assay. One of the most interesting outcomes of the extended interaction network of LC8 revealed a possible new function of the LC8 protein in the Hippo pathway through interaction with WWC and AMOT protein family members. The presented study significantly contributes to the better understanding of the functional and evolutionary properties of the LC8 interactome. Beyond this specific hub protein, it also offers general guidelines for the exploration of additional linear motif-mediated interaction networks.

## Results

### Scoring peptide segments based on known motifs

LC8 recognizes a short linear motif in its partner proteins. In this case, the binding involves both polypeptide chains of the homodimer LC8. However, to emphasize the similar binding mode to many single modular domains that also bind short linear motifs, LC8 is also referred to as a “binding domain” in this article. We assembled a manually curated database of LC8 interaction partners based on literature search, in which LC8 binding was verified at the motif level (see Materials and Methods and S1 Table). The final dataset contained 53 partners with 67 motif instances and covered multiple eukaryotic species and viruses. 40 motifs in 33 proteins belonged to human or could be directly mapped to a human protein based on close homology. The length of the core binding motif was taken as 8 amino acids (S1 Fig; for details, see S1 Text).

The ELM database describes the LC8 binding motif using the regular expression “^P.K.TQT”. From the 67 known motif instances, only 13 matched this regular expression, and 24 further motifs contained only the canonical “TQT” motif core. Considering human partners only, the corresponding numbers were 7 and 17, respectively. These numbers indicate that the definition in the ELM database is too restrictive to capture the majority of known instances. To better describe the common sequential properties of known LC8 binding sites, we used a position specific scoring matrix.

**Fig 1:**
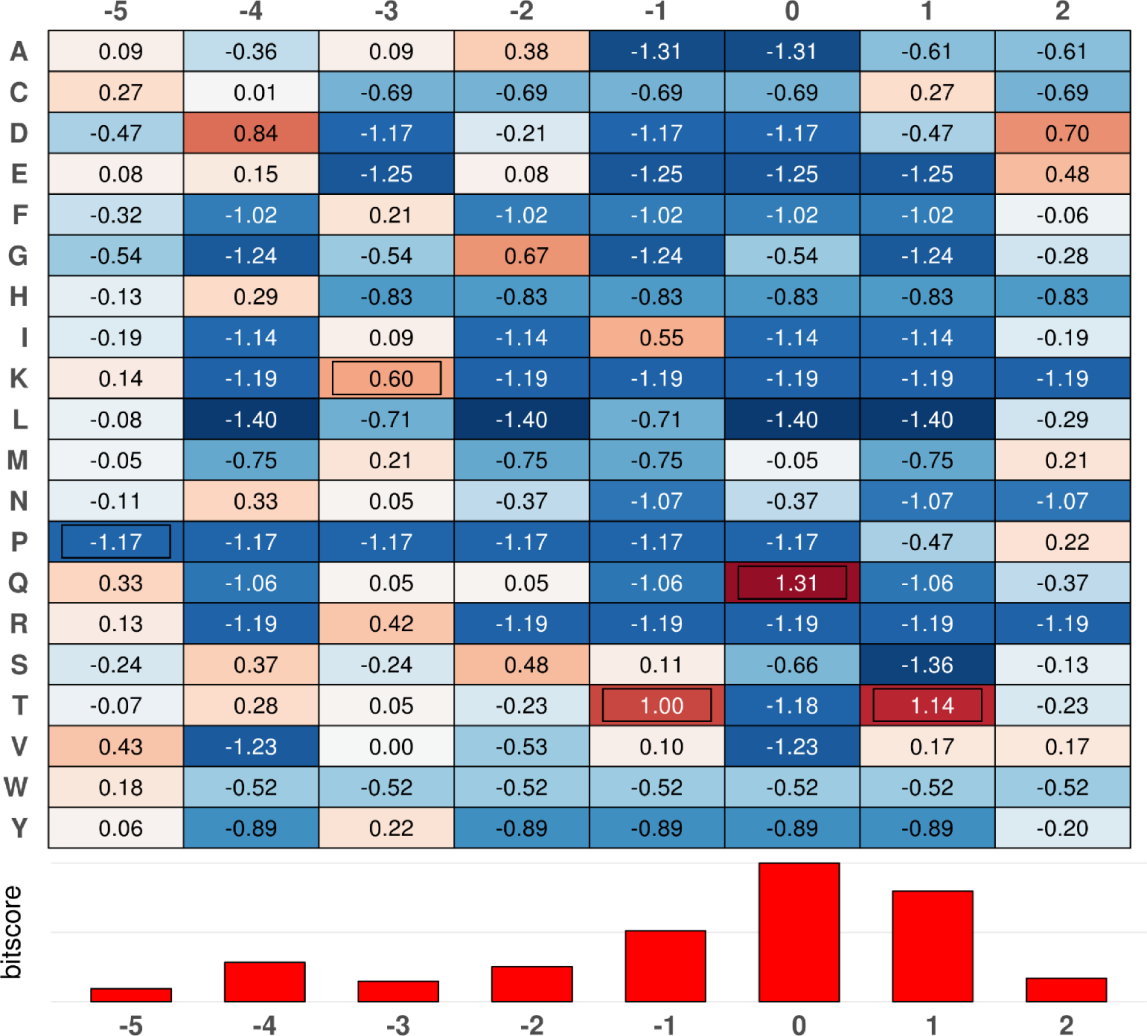
Position specific scoring matrix of LC8 binding motifs. PSSM score of each position of the binding motif numbered from the central glutamine of the canonical “TQT” motif core. Amino acids corresponding to the ELM definition are framed. Values are color scaled as a heat map ranging from blue (negative) to red (positive) centered on zero (white). The Shannon entropy of each column is shown below in bitscore.

The calculated PSSM is shown in Fig 1. Positive scores indicate amino acid residues that are favored at a given position. The PSSM clearly captures the frequent occurrence of the canonical “TQT” motif. However, with the exception of Gln in position 0, there are additional favored amino acids in every position. The strong preference for Lys at position -3 from the central Gln is not supported by the current collection of known partners, as neighboring positions have stronger preferences according to the bitscore. Using the obtained PSSM, we scanned the whole human proteome to score every overlapping eight amino acid long peptide segment (S2 Fig). As expected, all known LC8 binding peptides had a positive score. While only 2% of human peptides had a positive score, these still represented more than one hundred thousand cases. This indicates that on the one hand, positive PSSM scores are strongly associated with true binding motifs and can serve as a valid starting point for novel LC8-binding motif discovery. On the other hand, the very large number of initial motif hits underscore the need for that additional filtering steps.

### Establishing sequence features for filtering true binding partners

In order to establish additional filtering criteria, we gathered various predicted features of the PSSM-identified peptides. The methods we used included PFAM annotations [31], average disorder prediction scores (using IUPred, PONDR VSL2, Espritz and DISOPRED3) [32–35], average score to be part of disordered binding regions (using ANCHOR, MORF-CHIBI and DISOPRED3-BR) [35–37], and secondary structure prediction scores using PSIPRED [38]. At the protein level, information about predicted cellular localization [20,39] and the presence of coiled coil regions was also collected (See Materials and Methods). The various data for all known true positive motifs from human and other species are available at http://gerdos.web.elte.hu/data/LC8/known_results.html.

These various features can be associated with known motifs to different extents. The main challenge is to find the best tools and parameter settings that enable the prioritization of peptide segments that are the most likely to be biologically relevant motif hits in an optimal way. We introduced a metric based on weighted information gain derived from the Shannon entropy to globally attest the discriminatory power of each criterion. On a dataset containing 40 known human binding partners and 10,000 random human segments from the proteome with a higher than zero PSSM score, the weighted information gain was calculated for each criterion. The suggested measure enabled not only to rank the attributes in terms of their discriminatory power, but also to choose the best parameter settings. According to this protocol, the strongest criterion based on the information gain was the predicted intracellular localization, which was fulfilled by all known motifs, but only 85.53% of random peptides. The next strongest filtering criterion was based on PFAM annotations. Using annotation strictly based on the domain type PFAM families, it was possible to filter out 15% of random motifs, while retaining all known motifs. The third strongest criterion was based on PSSM scores. The highest information gain was achieved with the cutoff value of 3.3. This cutoff value was not met by only three known human motifs (PAK1, NRF1, MYZAP). At the next level, disorder prediction methods produced the highest information gain. Four methods were tested with different cutoff values corresponding to different false positive rates. The optimal choice was IUPred with the cutoff value of 0.42, which was fulfilled by all but one known motifs.

We also tested three methods (ANCHOR, MoRFchibi, DISOPRED3) [35–37] for predicting disordered binding regions also known as molecular recognition features (MoRFs). The information gain was much lower, compared to previous criteria. The optimal information gain was reached using the ANCHOR method with a cutoff value of 0.57. However, even this criterion would filter out 57.5% of the positive examples. An additional criterion considered was secondary structure prediction. Based on known structures of the complexes, the binding motif is expected to adopt a beta-strand conformation. Surprisingly, predicted beta-strands occurred in only four out of 40 cases, and even in these cases only very short segments were predicted to be in beta conformation. Helical segments were predicted in five cases, including MYO5A, which has been shown to have helical tendencies in the unbound form [40]. However, the lack of residues predicted to be in helix did not perform well as a filtering criterion as the majority of random hits were also predicted to lack regular secondary structural elements, and the overall information gain was below that of predicting disordered binding regions. Similarly, the presence of coiled coil regions in the partner proteins predicted by NCoils [41] did not have a strong discriminatory power either. As these criteria filtered out many known motifs, they were not included in our filtering protocol.

**Fig 2:**
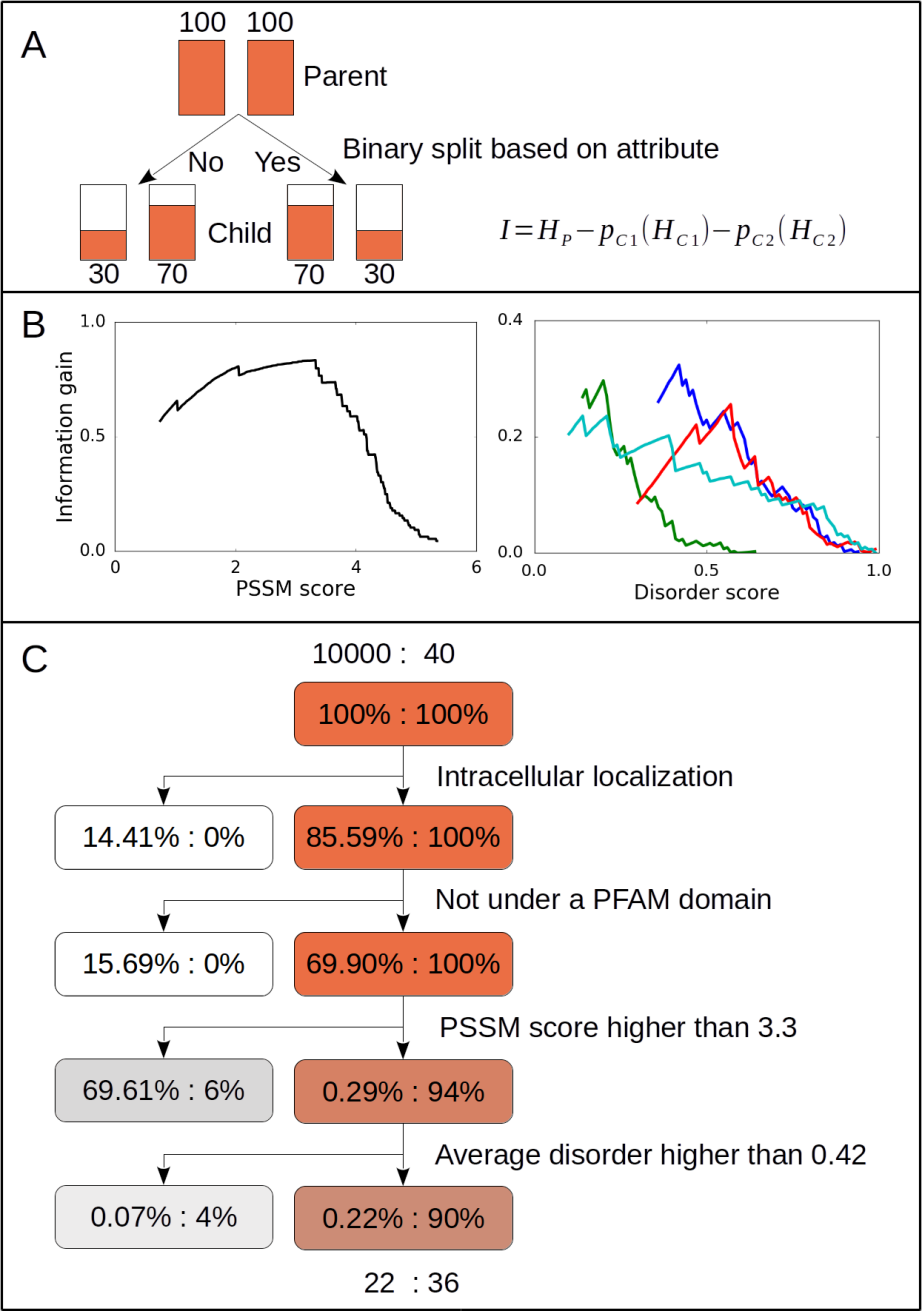
Filtering protocol to find true binding partners. (A) Schematic diagram of the binary filtering protocol we created utilizing information gain. A given attribute provides a binary split of the Parent group into the *C1* and *C2* child groups. The information gain (*I*) is then calculated as the difference of the Shannon entropy (*H*) of the Parent group minus the Shannon entropy of the Children groups weighted by their relative probabilities (*p*). These values were calculated over the dataset containing 40 known human binding partners and 10,000 random human segments from the proteome with a higher than zero PSSM score. (B) The information gain of the PSSM score (left panel) and four disorder prediction methods as a function of different cut-off values (right panel). The disorder prediction method used here were: IUPred (blue), Espritz Disprot (green) and VSL2 (red line), DISOPRED3 (cyan). Optimal cut-off values were obtained from the cut-off value corresponding to the maximum of the information gain, yielding 3.3 for the PSSM score, and 0.42 for IUPred disorder prediction score. (C) The outline of the final filtering protocol indicating the number of elements and percentage of cases in each Child group with the applied binary split.

The resulting filtering protocol is shown on Fig 2, indicating the proportion of random hits and known motifs that were eliminated at each step. By the stepwise application of the four filtering steps, a drastic reduction of random hits could be achieved, while still retaining the majority of known motifs: 90% of known motifs were kept while 99.78% of random hits were filtered out (i.e. only 0.22% was kept). By applying the filtering protocol for the complete human proteome, 335 candidate motifs remained. We carried out a 3-fold cross validation to measure the generality of the obtained filtering protocol (see Materials and Methods). On average, we were able to correctly categorize 34 examples from the 40 experimentally validated human binding motifs. This was only marginally worse compared to using the complete database where 36 motifs were categorized correctly. The cut-off values for the PSSM and IUPred scores also did not change largely. This indicates that the protocol is robust and can correctly describe the general attributes of the binding event between LC8 and its known binding partners.

### Evolutionary conservation of motif hits

Given the extreme conservation of LC8, it is of special interest how the interaction motifs are conserved in partner proteins. To study this, we generated multiple sequence alignments of orthologous proteins harboring known LC8 binding motifs and categorized them into 5 taxonomic groups: Mammalia, Vertebrata, Metazoa, Fungi and Eukarya. The conservation at the level of protein and motif was tested based on the obtained alignments in each taxonomic group. The definition of motif conservation applied here depends on the PSSM score of the motif (see Materials and Methods). Consequently, this approach cannot be applied to motifs that significantly differ from canonical motifs, i.e. their binding motif had a PSSM value below the threshold. These three examples were excluded from the analysis. Nevertheless, this strict criterion ensured that not only the general sequence similarity is maintained, but also the similarity to known human LC8 motifs is preserved.

**Fig 3:**
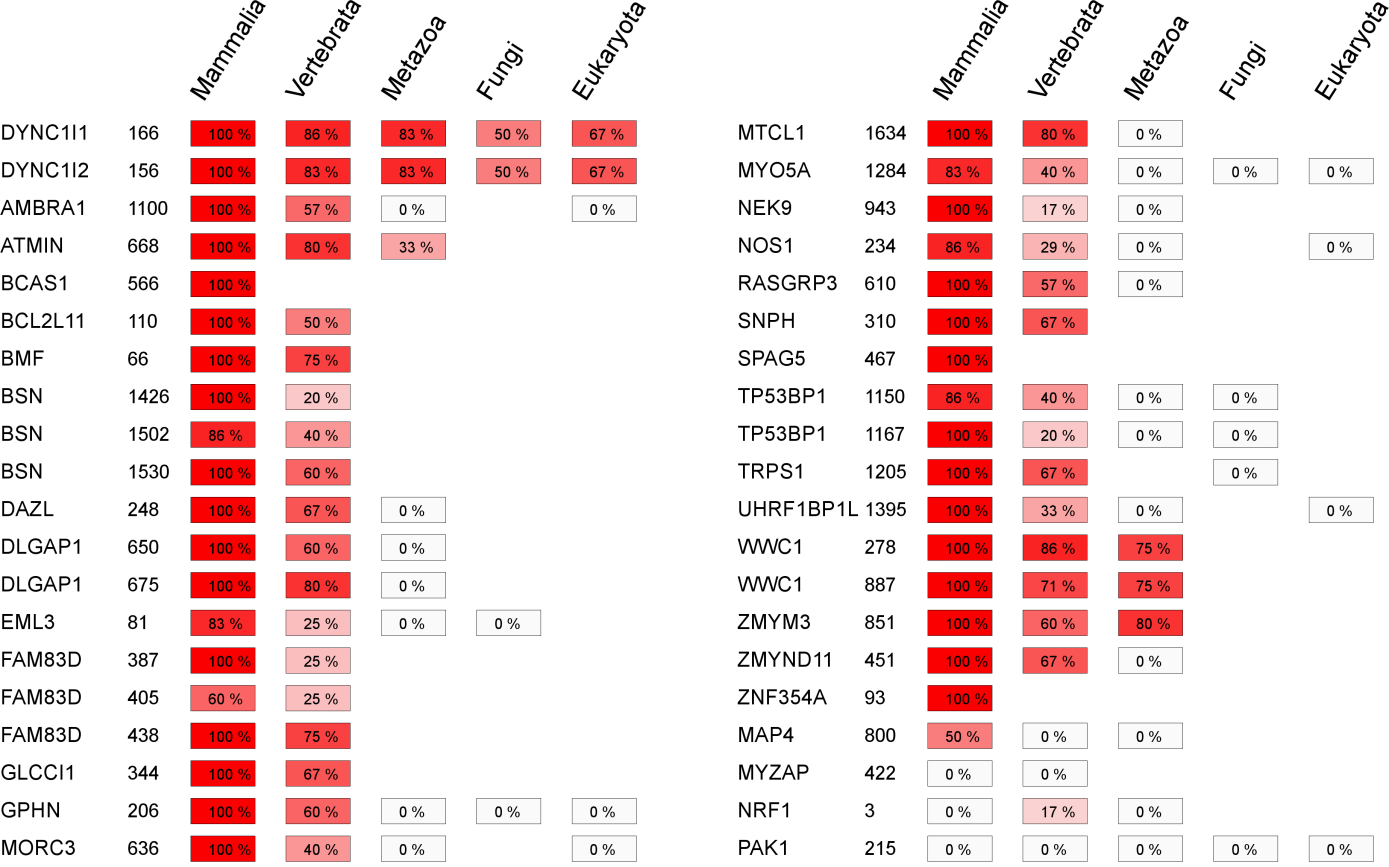
Summarized evolutionary conservation results of known human LC8 binding partners. Protein names and motif start positions are indicated in the first and second columns. The colored boxes represent the presence of orthologues of the known partner at different evolutionary levels. The percentages and colour scheme of the boxes show the PSSM based motif conservation across all species. Conservation values increase from white (low motif conservation) to red (high motif conservation).

The results of the conservation analysis for the known human motifs are presented in Fig 3. Among the known partners, the LC8 binding sites located within dynein intermediate chains (DYNC1|1, DYNC1|2) exhibited the most pronounced conservation, spreading across a wide range of eukaryotic species (e.g. starlet sea anemone, *C*. *elegans*, slime mold). In contrast, other motifs identified in human or other mammalian species were generally not conserved beyond Vertebrata (e.g., BMF, DLGAP1, MTCL1). In the majority of cases, not only the LC8 binding motif but the complete protein was lost beyond this evolutionary distance (e.g. BMF, BSN, SNPH). Similarly, limited conservation of LC8 motifs was observed for binding regions experimentally verified in other species (S3 Fig). For example, the EGL protein from *Drosophila melanogaster* had orthologues at each level, but its binding motif showed conservation only in metazoan species. While the centriole duplication functionality is conserved across metazoan species, the LC8 binding protein ANA2 involved in this process is specific to *Drosophila* species [42]. Lowering the PSSM cutoff value used to define the conservation of the motifs perturbed the results only in three cases: NRF1, FAM83D and MYO5A, but had no major impact on the overall trends (S4A and S4B Fig). We can conclude from this analysis of motif conservation that while the LC8 binding interface is highly conserved, known partners and especially the binding motifs located within them are significantly less conserved.

We tested the specific conservation pattern of motif binding sites using the SLiMPrints algorithm [19]. This method identifies short stretches of residues within disordered regions that show high relative conservation compared to their flanking region as calculated from multiple sequence alignments of orthologous sequences. In our dataset, 13 out of the 40 known human motifs exhibited high relative conservation, which is only 32.5% of all cases. This suggests that the approach based on the island-like conservation identified by SLiMPrints has a limited ability to highlight putative functional binding motifs and overall it is not a good filtering criterion, as it misses the majority of functional motifs.

Here, we suggest an alternative filtering criterion based on the observation that known motifs are generally conserved within their own taxonomic group even if they lack motif level conservation over wider evolutionary distances. For the majority of the cases, known motifs in human sequences were conserved in at least 80% of mammalian species. The exceptions included only the three cases that had a PSSM value below our cutoff, and two additional examples (the MAP4 motif and the second motif of FAM83D at position 405). In contrast, randomly chosen motif hits located within disordered regions generally did not possess this property. Applying this discriminatory technique (see Materials and Methods), we could filter out 163 of the possible candidate peptides, reducing the number of candidate motifs to 172. Therefore, for human sequences the conservation within mammalian species can be used as an efficient filter to further reduce likely false positives.

### Integrating protein-protein interaction data

The predicted motif hits were also analyzed in relation to experimental data in PPI databases. Known interaction partners of LC8 were collected using the integrative PSICQUIC approach [43], which enabled us to search multiple databases simultaneously and to group the experiments based on detection methods and association types (see Materials and Methods). Altogether, 381 LC8 interaction partners were collected (S2 Table). Several of these interaction partners were supported by multiple lines of evidence, with a total of 782 independent experiments. Known motif partners were well represented in current PPI databases, with only three missing out of these 33 partners. The comparison of interaction partners collected from PPI databases with those that contained known motifs indicated that there is no single criterion that can identify biologically relevant interactions from candidates in PPI databases. However, PPIs from low-throughput and direct experiments are more likely to be biologically relevant, especially when they are supported by multiple independent measurements (see S5 Fig).

**Fig 4:**
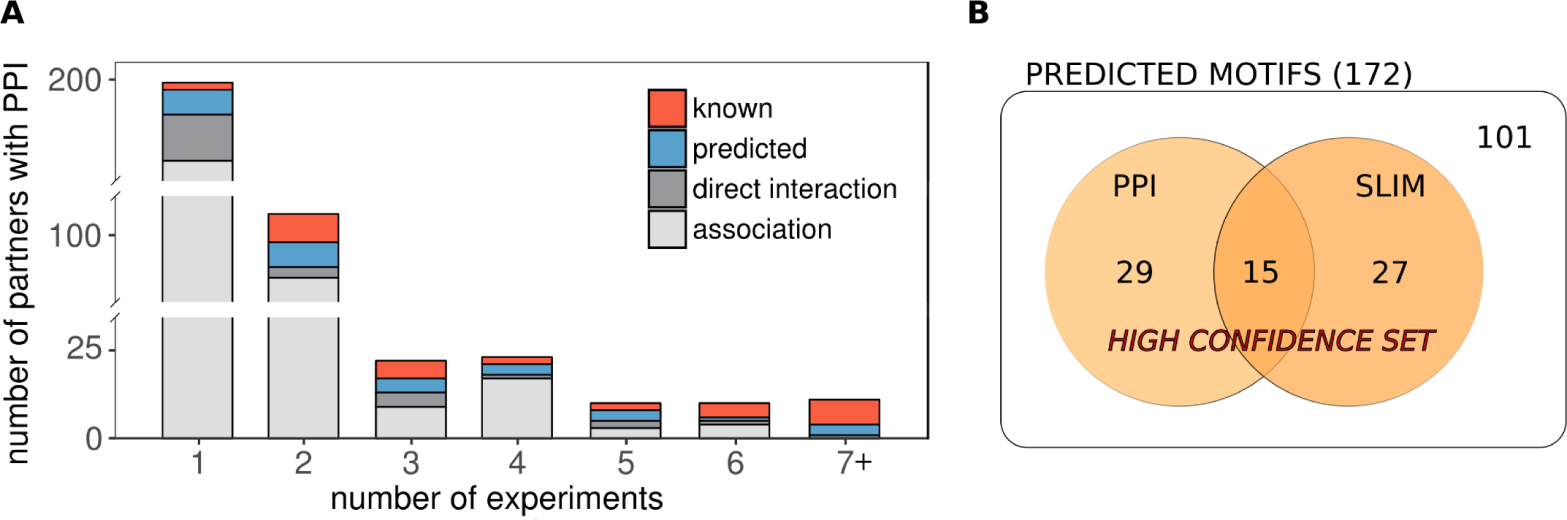
Distribution of LC8 partners and predicted motifs. (A) Distribution of LC8 interaction partners from PPI databases over the number of experiments. Partners that contain neither known nor predicted motifs are shaded grey according to the interaction type. (B) The composition of the high confidence prediction set shown as a Venn diagram. PPI: motifs in partners that appear in PPI databases. SLIM: motifs that show island-like conservation with the SLiMPrints method.

Overall, there were a large number of potential partners recorded in PPI databases (323) that contained neither known nor predicted LC8 binding motif instances. By looking at the distribution of partners over the number of supporting experiments, we can see that partners with known and predicted motifs were largely evenly distributed, similarly to the partners detected by direct methods (Fig 4A). Known and predicted motifs dominated in partners with at least 7 supporting measurements, with a single exception corresponding to KANK2, in which no motif-like segments could be identified. In contrast, most of the partners without a likely motif candidate were detected by only a single, indirect method. Although it cannot be ruled out that some of these interactions represent an alternative binding mechanism to LC8, the large number of PPIs that are not compatible with the existing binding mode underlines that data from PPI databases should be treated with caution as they may contain several false positives.

Among the 172 predicted motifs in 152 proteins that satisfied our filtering criteria, there were 43 novel motif instances predicted by our pipeline that had corresponding PPI data. While most of these interactions were based on indirect, high-throughput experiments, many of them were supported by multiple measurements. 8 of these motifs represented additional instances in proteins that already contained an experimentally verified binding motif. In 35 cases, the likely binding region for LC8 could be identified for proteins whose interaction were studied only at the level of the protein (see S2 Table). Furthermore, the presence of PPIs lends support to the biological relevance of the predicted motifs in these cases. By considering motif hits located in proteins with corresponding PPI data, we created a dataset that contained a list of high confidence motif instances for LC8. In addition to PPIs, the presence of island-like conservation was also taken as an indication of likely motif hits. Altogether, we collected 71 high confidence motif instances, supported by PPI data (29 cases), the presence of island-like conservation (27 cases), or both (15 cases) (Fig 4B). Using these complementary information, the number of interaction partners of LC8 could be practically doubled (S3 Table). The high confidence motifs are available at http://gerdos.web.elte.hu/data/LC8/HUMAN/high_confidence_hits.html.

**Fig 5:**
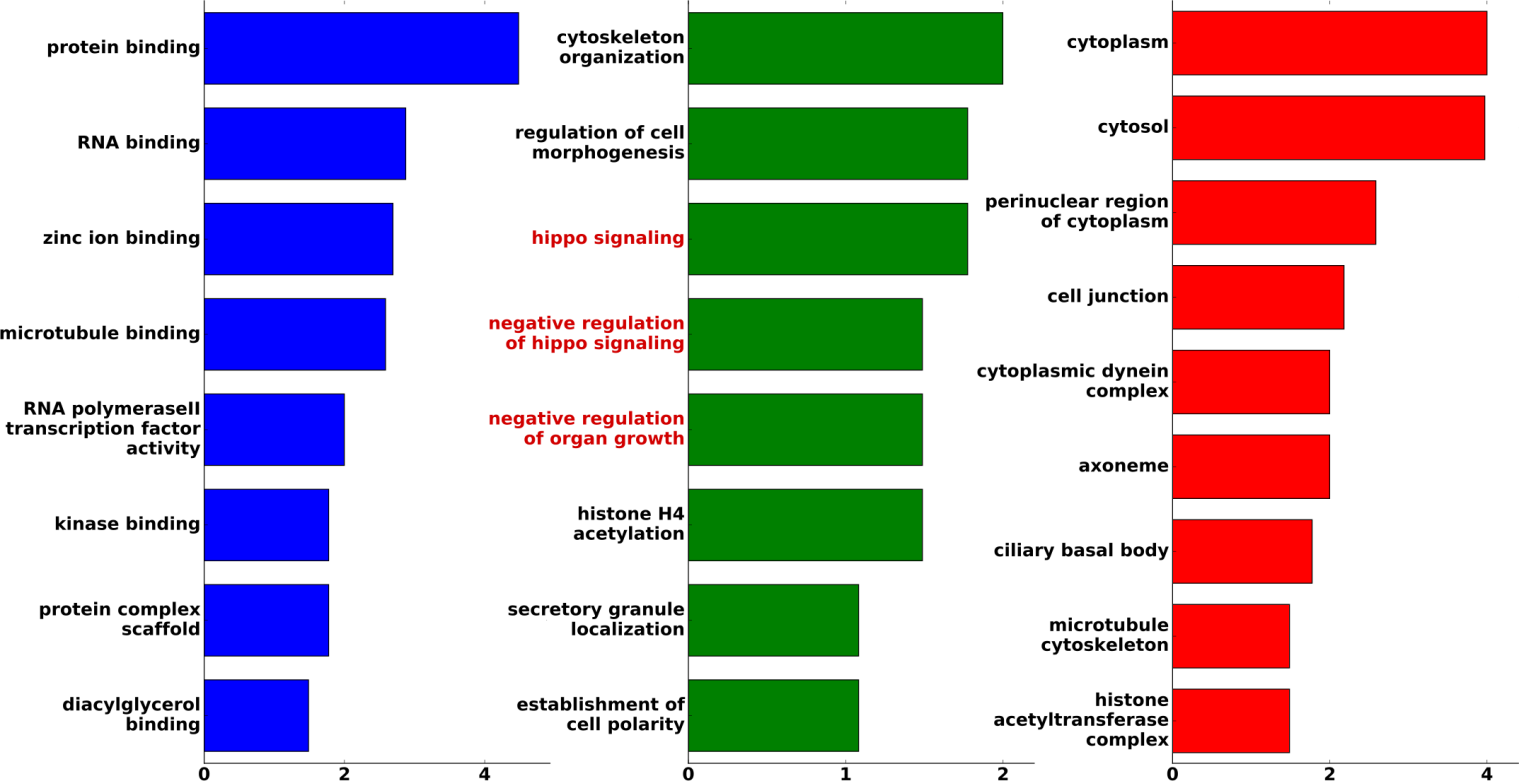
GO enrichment in the high confidence set of LC8 binding partners. Molecular Function (blue), Biological Process (green) and Cellular Component (red) categories that are enriched in the high-confident LC8 binding partners compared to human background. The x-axis represents the log-odds ratio of each enriched GO category. Process names related to the Hippo pathway are colored in red.

The high confidence set together with the already known motif hits was used to carry out a GO enrichment analysis against the human proteome with the DAVID server [44]. The analysis showed an enrichment of multiple functionalities previously associated with LC8, such as cytoskeletal organization, microtubule binding or cell morphogenesis (Fig 5). Besides the already known activities, the enrichment analysis revealed a highly over-represented, yet previously undiscovered function of LC8 that links the protein to the regulation of cell and tissue growth, and in particular, to the Hippo pathway. This result suggests that LC8 might play a critical role in the regulation of the Hippo pathway.

### High confidence motif hits establish a role of LC8 in the Hippo pathway

In order to understand how LC8 is connected to the Hippo pathway, we took a closer look at the relevant partners. The motif hits highlighted two families involved in the upstream regulation of the Hippo pathway, the WWC (WWC1, WWC2 and WWC3) and angiomotin (AMOT, AMOTL1, AMOTL2) families [45,46,47]. The WWC family member WWC1, also known as KIBRA, was shown previously to interact with LC8 [48], and two binding motifs were also identified, located at positions 278 and 887 (highlighted in Table 1) [49]. However, no binding sites have been previously identified in the other two members of the family, WWC2 and WWC3. Interactions between LC8 and the AMOT family members were previously reported in high-throughput studies to be LC8 interactors [50], but the binding motif has not been located in any of these proteins as of yet. Here, we identified the likely LC8 binding regions in all WWC and AMOT family members and analyzed their evolutionary conservation. The binding of these peptides was verified and quantitatively characterized by SPR (Table 1).

**Table 1:** Experimentally validated LC8 binding motifs in the Hippo pathway.

Gene name	UniProt Accession	Start	Binding motif sequence	K_d_(μM)±SD
WWC1*	Q8IX03	278	LDVSSQTD	0.45±0.03
WWC1*	Q8IX03	887	VDKETNTE	4.00±0.50
WWC2	Q6AWC2	282	LDAGSQTS	32.7±2.97
WWC2	Q6AWC2	962	VDKETNTD	17.6±0.54
WWC3	Q9ULE0	208	CDAGSQTD	5.34±0.18
WWC3	Q9ULE0	867	VDKETNTE	3.50±0.15
AMOT	Q4VCS5	860	RDCSTQTE	1.20±0.04
AMOTL1	Q8IY63	873	KDSSTQTD	1.55±0.04
AMOTL2	Q9Y2J4	724	RDGSTQTE	1.98±0.19

*The two WWC1 motifs were validated in a previous study [49]

**Fig 6:**
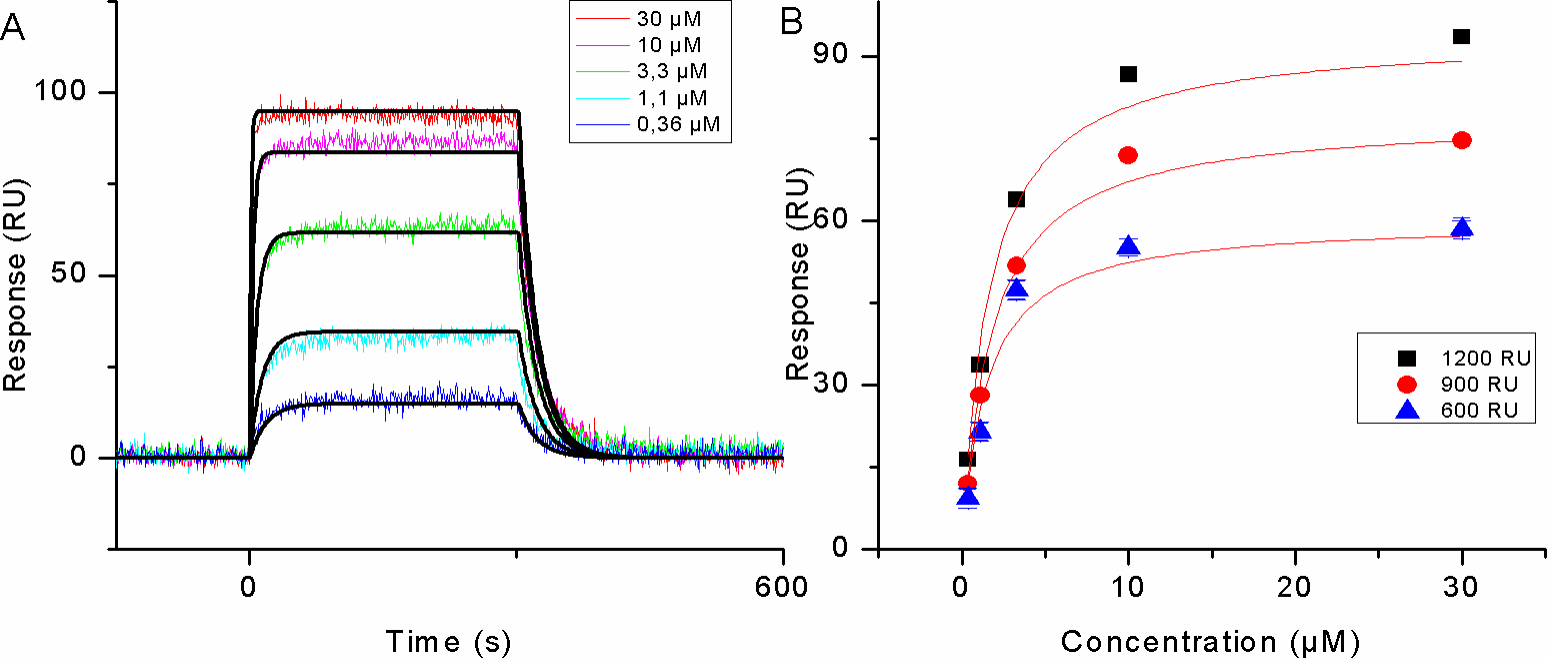
SPR analysis of the LC8 –AMOTL2 interaction. (A) AMOTL2 peptide was injected to His_6_-LC8 immobilized on Tris-NTA sensor chip as described in Materials and Methods. Sensorgrams derived from five different analyte injections are indicated with colored lines. Black lines show the global fit of the experimental data to a 1: 1 Langmuir model. (B) Equilibrium analysis of the LC8 –AMOTL2 interaction at different LC8 immobilization levels.

The list of putative LC8 binding peptides identified by our protocol are shown in Table 1, together with the measured binding constants, which confirmed the binding of the selected peptides to LC8. As an example, results are shown for the motif in AMOTL2 in Fig 6. Although the apparent K_d_ values indicate weak interactions, these binding affinities are similar to other LC8 interactions [25,20], and in biological settings they can increase in strength due to avidity caused by dimerization [30]. Besides the canonical TQT motifs these peptides also contain SQT and TNT motifs. Additional peptides that further expand the repertoire of compatible amino acids at various positions were also tested for their binding to LC8 (S4 Table).

**Fig 7:**
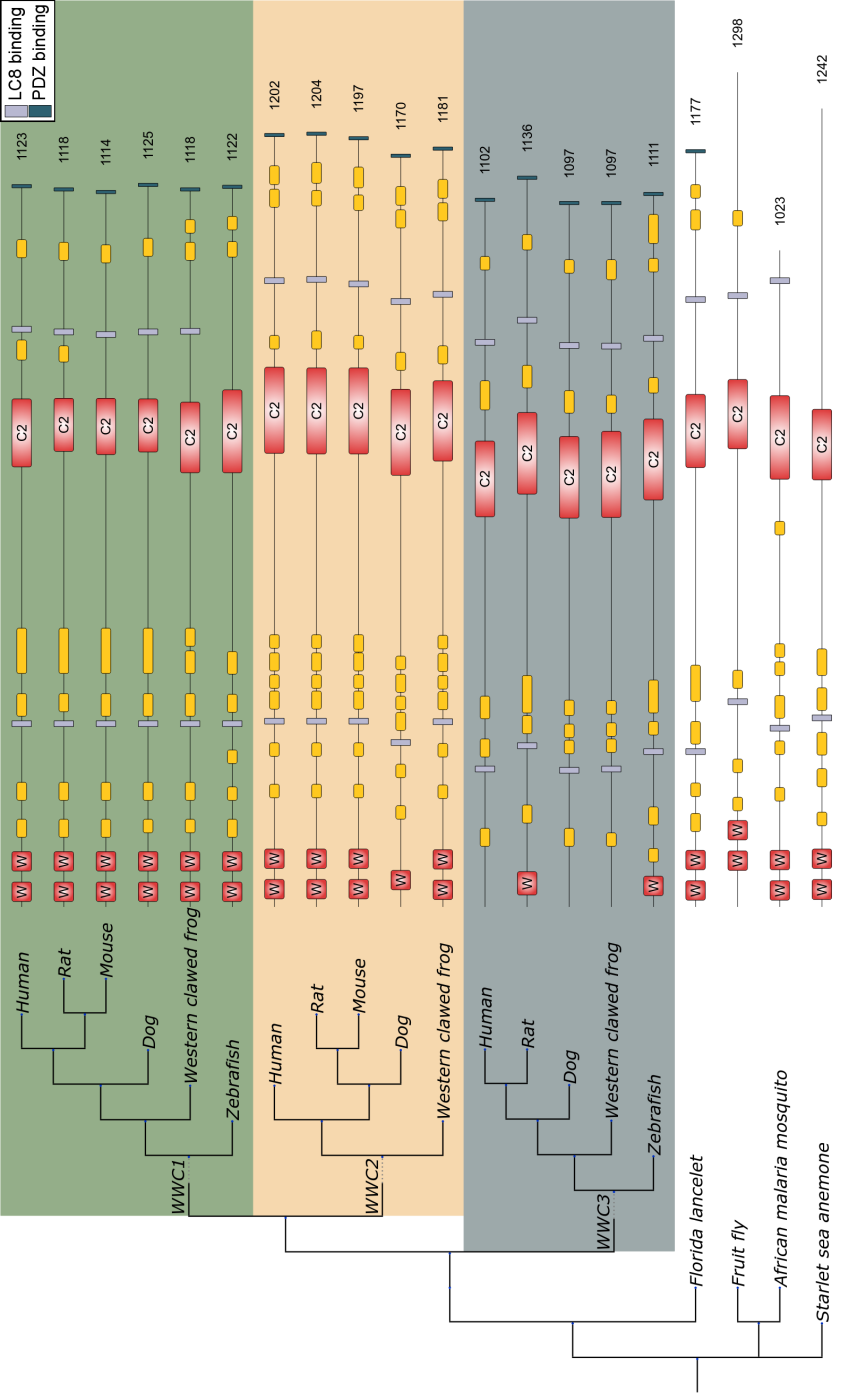
Structural organization and phylogenetic tree of the WWC family members. The tree branch lengths do not represent real values. The subtrees of the three vertebrate WWC paralogs are highlighted by colored rectangles. Red and yellow boxes indicate the domains and coiled coil regions, respectively. Small purple and dark green boxes mark the location of the LC8 and PDZ binding motifs, respectively.

**Fig 8:**
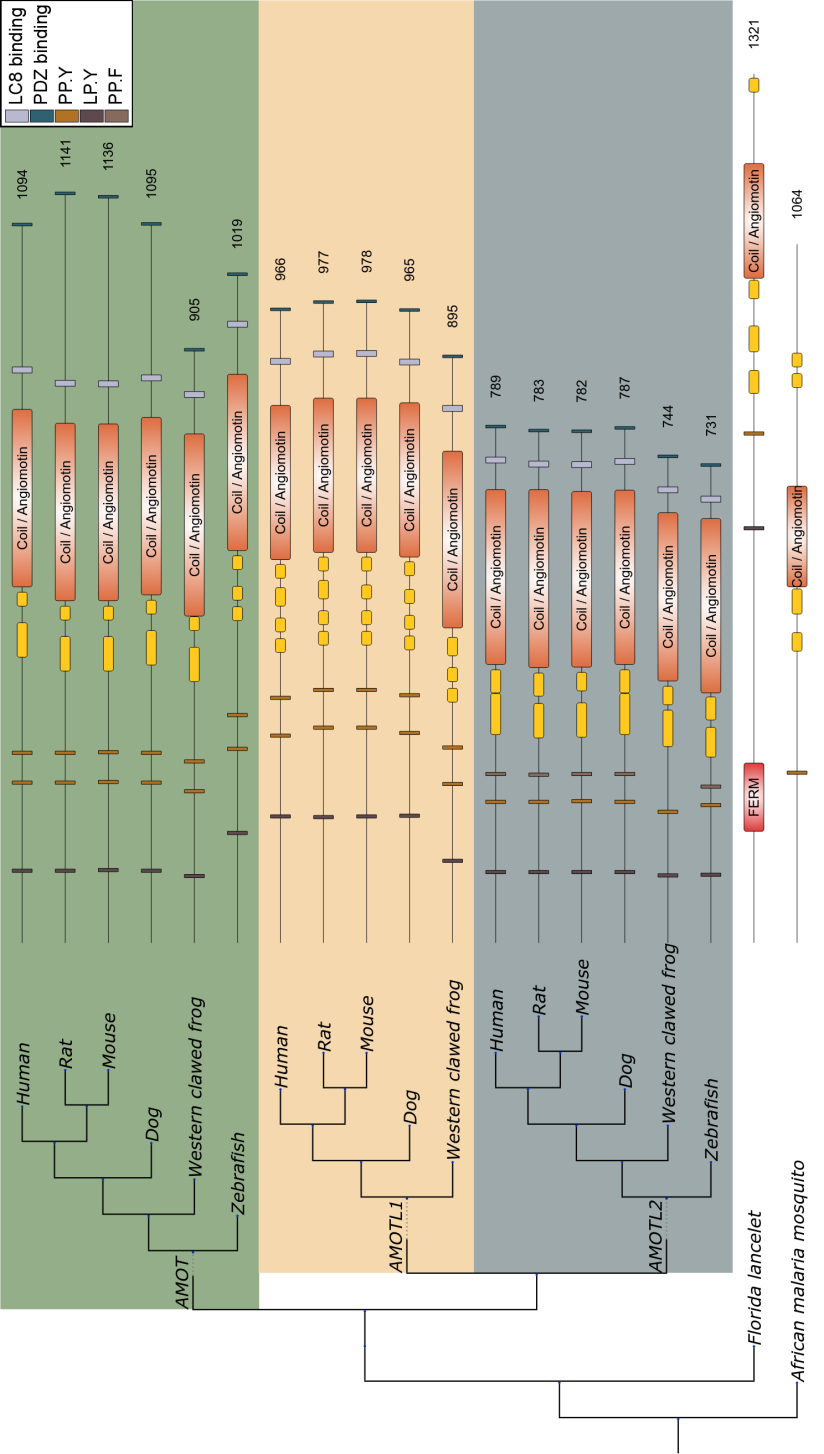
Structural organization and phylogenetic tree of the AMOT family members. The tree branch lengths do not represent real values. The subtrees of the three vertebrate AMOT paralogs are highlighted by colored rectangles. The angiomotin PFAM family was merged with overlapping coiled coil regions and indicated as angiomotin/Coil domain in orange. Other coiled coil regions and domains are colored yellow and red, respectively. Small boxes mark the location of linear motifs: brown (dark, light, medium)—WW binding, purple—LC8 binding, and dark green—PDZ binding motifs.

In order to properly assess the conservation of LC8 binding motifs in WWC and AMOT family members, we traced the evolutionary history of these proteins and their identified binding regions and additional functional modules. Almost all vertebrate species have three paralogs of both the WWC and AMOT family members (Figs 7 and 8). For both families, the multiple paralogs observed at the level of vertebrates could be traced back to a single common ancestor gene, as shown in the case of Florida lancelet. KIBRA orthologs were detected beyond the level of chordates, not only in arthropods, but also in cnidaria (Fig 7). AMOT was also present in arthropods, but it was missing in the fruit fly (Fig 8). The most likely explanation for this is that the angiomotin was originally present in all bilaterian animals, but was lost relatively recently in the dipteran lineage that includes Drosophila [51]. Altogether, the WWC family members seem to have a more ancient evolutionary origin compared to angiomotins.

Both WWC and AMOT family members showed a highly conserved domain and linear motif organization across a wide range of species (Figs 7 and 8). While most LC8 binding motifs verified in human sequences are not conserved beyond vertebrates, KIBRA and the other WWC family members represent an important exception in this regard. The two LC8 binding motifs of this protein family are well conserved in all three paralogs in vertebrates, lancelet and fruit fly. Furthermore, one of the motifs is also present in a sea anemone, matching the strong evolutionary conservation of the WW and C2 domains, the defining functional modules of this family (Fig 7). The AMOT family members contained a single LC8 binding motif, which seems to have emerged more recently. While orthologous sequences could be detected in non-vertebrate species based on the conserved coiled coil region together with the angiomotin domain, these sequences lacked the LC8 binding motif (Fig 8). The LC8 motif, similarly to the PDZ and WW binding motifs, has become conserved within vertebrate species. In both of these families, the redundancy and conservation underline the functional importance of LC8 binding motifs in the upstream regulation of the Hippo pathway.

## Discussion

In this work, we established a systematic filtering protocol that can be used to expand the interaction network of a given linear motif binding domain with high confidence motif hits and reduce the number of random motif matches. While similar bioinformatic pipelines have been used before [14], the optimality of the applied filtering steps could not be guaranteed or even assessed due to the lack of appropriate measures. Here, we offered a solution to this problem by introducing a decision tree-like filtering procedure together with the weighted information gain that enabled the construction of an optimal bioinformatic pipeline. The presented approach was applied to expand the interaction network of LC8, a highly conserved eukaryotic hub protein that binds its partners via a specific linear motif [22]. By combining our motif filtering protocol with data collected from protein-protein interaction databases and the information on the presence of island-like conservation, we created a dataset of 71 novel high confidence motif instances (S3 Table). These novel binding sites significantly enriched the interaction network of LC8 with novel partners and highlighted a previously unknown, important function of LC8 in the Hippo pathway.

A list of predicted LC8 binding motifs was also created in an earlier work [20]. In this case, the PSSM was calculated based on a library of binding motifs evolved through phage selection instead of using the collection of naturally occurring motif instances. Although some of the filtering criteria, like IUPred based disorder prediction or intracellular localization were used in both studies, the details of implementation differed significantly, including the optimal cutoff for the PSSM score. The comparison of the two sets showed that among the top 83 hits identified based on the in vitro evolution and absent from the list of known naturally occurring motifs, 58 (70%) were also uncovered by our presented motif discovery pipeline. However, our evolutionary filtering criteria reduced this overlap to 38 (46%). While to some extent, the limited overlap could be due to the technical differences in the implementation, a more appealing explanation is that binding motifs selected by phage display are optimized for binding strength, while the sequence of naturally occurring motifs were shaped by various evolutionary requirements in the cell, from which strength is just one, and not necessarily the dominant constraint. Despite these differences, in vitro evolution by phage selection [20,52] represent a complementary approach to predict potential motif instances for specific binding domains and to explore the binding preferences of such domains.

While the filtering protocol implemented here is specific to LC8, the presented work also has important implications beyond this system. We suggested here a general framework to find the optimal selection of methods for the filtering steps. The importance of this optimization can be best demonstrated through the example of disorder prediction methods. Several methods, such as DISOPRED3, PONDR VSL2 or ESpritz Disprot [33–35], that were tested in this work, perform better on specific datasets of ordered and disordered proteins [53–55]. Nevertheless, IUPred achieved the best results for this specific problem, supporting the choice of this approach in motif-centric application of protein disorder [4,14,19]. The pipeline applied here also enabled us to identify features that had limited discriminatory power and therefore were not incorporated into the current pipeline. For example, all known LC8 binding motifs adopt a β-strand conformation upon binding, but the current tools are not capable of capturing this property efficiently. Additional features, such as predicted disordered binding regions/MoRFs, the presence of coiled coils or island-like conservation also had limited discriminatory power. As these properties are clearly associated with at least a subset of LC8 binding partners, they highlight potential areas where more efficient methods are needed.

The analysis of evolutionary information was also highly valuable to increase the biological relevance of potential functional sites, but also provided some unexpected results. LC8 is ubiquitous in all eukaryotes, presenting a highly conserved binding interface for its interaction partners, which is practically identical from Drosophila to human. One of the most well-characterized interaction partner of LC8, the dynein intermediate chain is also conserved, at least in the animal kingdom. Therefore, it was surprising to find that most of the other known interaction partners of LC8 showed limited evolutionary conservation at the motif level. The overwhelming majority of both known and predicted motifs in the human proteome could not be traced beyond vertebrates. These results are in agreement with the evolutionary plasticity of linear motifs, as SLiMs can be introduced or eliminated by a few point mutations, or created ex-nihilo [56]. However, the lack of motif conservation was unexpected in the light of the strong evolutionary constraints acting upon LC8. Nevertheless, signs of purifying selection could be detected for the binding motifs over smaller evolutionary distances. In a subset of cases, the SLiMPrints method [19] was able to detect that motif residues showed higher relative conservation compared to their flanking regions. However, this approach failed to highlight many validated binding motifs, either because of problems with alignments, a strong overall conservation or the lack of the sensitivity of the method. In our experience, a more efficient filtering criterion was the conservation of motif residues within mammalian species. This criterion was true for nearly all verified human binding motifs, but was not met by most of the random motif hits that were located within the evolutionary variable disordered regions. Therefore, it is a highly efficient filtering approach. Nevertheless, we understand that by filtering based on mammalian-level conservation comes with a risk of losing some instances that are conserved only in a smaller taxonomic unit within mammalia, e.g. in primates.

The expanded interaction network of LC8 highlighted novel functions and drew attention to the potential role of LC8 in the Hippo pathway which controls organ size and cell growth by regulating cellular proliferation and apoptosis [57]. In our high confident hits, two putative LC8 binding partner families, the WWC and the angiomotin family were identified. Both families are tightly connected to the Hippo pathway and are known to be involved in its upstream regulation pathway in multiple ways [58]. WWC and AMOT family members can act either by activating the core kinase modules or by forming complexes with YAP/TAZ sequestering them at cell-cell junctions and preventing their nuclear access [59–64]. Previously, the interaction between WWC family members and LC8 has been established at the protein level [65], but the binding motifs were identified only in WWC1/KIBRA [49]. In addition, a study of the protein interaction network of the mammalian Hippo pathway also revealed interaction between AMOT proteins and LC8 [66], confirmed by additional large-scale studies [67,68]. A recent work established a connection between LC8 and the Hippo pathway in Drosophila [69]. In the present work, the LC8 binding sites were experimentally verified both in WWC and the AMOT protein families and were shown to be evolutionarily conserved.

The verification of binding sites established a direct connection between LC8 and the Hippo pathway through the WWCs and AMOT family proteins, nevertheless, the exact role of these interactions in the regulation of Hippo pathway is still unclear. Recently, LC8 was suggested to promote dimerization of partially disordered proteins by stabilizing their coiled coil regions [25]. This model was based on detailed characterization of the complex formation of several scaffolding proteins, such as Swallow, dynein intermediate chain DYNC1|1 and GKAP [70]. However, there are still open questions regarding the regulation of binding of LC8 to its partners. In agreement with the general model for LC8 function, both families contain coiled coil (CC) regions and the dimerization of both AMOT [71,64] and WWC family members [72] might be necessary for their scaffolding activity. While the functional role of the CC region in the WWC family has not been explored, the CC regions of AMOT family members were shown to form interactions with various components of the Hippo pathway and influence their activity and localization [59,63,73,64]. Therefore, the potential stabilization of coiled coil regions driven by LC8 could also play important roles in regulation of the Hippo pathway. However, the LC8 function related to intracellular trafficking cannot be ruled out entirely either, as both KIBRA and AMOT proteins alternate between cytoplasmic and nuclear locations depending on various signals [39,74]. While LC8 might provide an important additional layer to the regulation of the Hippo pathway, further investigations are needed to understand how the interaction of LC8 affects WWC and AMOT proteins in the Hippo pathway and in other processes.

In conclusion, the novel binding partners presented in this work provide exciting new opportunities to study how the binding of LC8 influences the stability and binding properties of various proteins. In addition, a general framework was also proposed here for the optimization of the linear motif filtering. While the protocol was applied specifically to LC8 and takes advantage of its relatively well-characterized interaction network with over 60 known linear motif instances, it can be applied in a similar way for other linear motif binding domains for which similar number of partners have been identified. In a more general sense, the novel motif filtering protocol further underlines that computational approaches can complement large-scale experimental studies of linear motif binding systems, and advance our understanding of how these relatively weak, transient interactions contribute to highly dynamic, complex regulatory processes in the cell.

## Materials and methods

### Computational filtering procedure

We collected 76 LC8 binding motifs from the literature, however, upon further investigation we rejected 9 of them due to the lack of motif level evidence. The resulting list is given in S1 Table (for details, see S1 Text). The two vertebrate isoforms of LC8, DYNLL1 and DYNLL2 were not discriminated in this study. From the collected 67 binding motifs we generated a non-redundant set using the CD-HIT suite tool [75]. The remaining 62 motifs were aligned and used to construct a PSSM. The values in the PSSM matrix capture the preferences of each amino acid for over-representation or under-representation at each position in the binding motif. The elements of the PSSM (P_i, j_) were expressed as the log-odds score of amino acid frequency in each position in the known binding partners divided by the background frequency:

$$P_{i,j}=log(\frac{A_{i,j}}{D_{i}})$$

where *A*_*i*, *j*_ is the frequency of amino acid *i* at position *j* in the alignment of known binding partners, and *D*_*i*_ is the background frequency of amino acid *i*. The background frequency was derived from the frequency of amino acids in the UniProt Eukaryotic proteome. As not every amino acid was present in each position in the known partners, we incorporated a pseudo-count correction to account for these zero occurrences.

$$P_{i,j}=log\left(\frac{\frac{A_{i,j}+\frac{B}{20}}{m+B}}{D_{i}}\right)$$

Where *B* is the pseudocount with a value of 5, as suggested in previous works [76], *m* is the number of sequences, and 20 is the number of amino acids.

Additional sequential features were calculated for each known binding partner to identify the attributes of true binding events. The binding partners were analyzed using more than 20 different tools, to predict disorder content (IUPred, PONDR VSL2 and DISOPRED3) [32,33,35], the tendency to be involved in disordered binding regions (ANCHOR, MoRFchibi and DISOPRED3-BR) [35–37], secondary structure and subcellular localization predictions. For each potential binding motif, position-based numerical values were averaged. The subcellular localization of a motif was predicted by a combination of methods, and only putative motifs with predicted intracellular localization were considered to be accessible for interaction with LC8. A motif was considered to be intracellular if it contained no predicted signal sequence according to the SignalP predictor [77]. Proteins with a predicted signal sequence were further analysed using PHOBIUS [78] for transmembrane topology and the motif was categorized according to its result to either be in intracellular (INT), transmembrane (TM) or extracellular space (EXT). PFAM identifies conserved sequence families, which can be of different types, including domains, families, motifs, repeats and also intrinsically disordered domains [31]. Each motif was categorized as Domain or Family if it overlapped in at least one position with a respective PFAM annotation [31]. Secondary structure predictions were carried out using PSIPRED [38]. Proteins were checked with NCOILS to predict coiled-coil regions [41]. The calculated features can be found at http://gerdos.web.elte.hu/data/LC8/known_results.html.

A protocol was generated to find the optimal filtering criteria based on these sequence features. Due to the limited number of positive examples, the full tree space was not explored, instead, optimal filtering methods and the best parameter settings were selected globally. For this, we defined two sets, one from the known human motifs, and one from 10,000 randomly selected peptides from the human proteome with PSSM scores above 0. Various attributes were calculated for each motif in both sets. We sought to find attributes that can keep all or most of the known motifs while discarding the largest number of the randomly selected peptides. The optimization was guided by using the information gain theory derived from the Shannon entropy.

$$I=H_{p}-p_{c1}(H_{c1})-p_{c2}(H_{c2})$$

Where *I* is the information gain, *H*_*p*_ is the Shannon entropy of the parent group, *c*_*1*_ and *c*_*2*_ are the child groups on a specific split, and *p* is the probability of an attribute in the given group. During the entropy calculations, the number of occurrences with a specific attribute were normalized with the sum of the examples to weight the imbalanced sets. The information gain reaches its theoretical maximum in the ideal case, which results in a clean set, e.g. when a criterion is true for all entries in the known motif set. In these cases, the best attribute was defined as the one that filtered out the largest number of random peptides.

We validated the filtering protocol using a 3-fold cross-validation. The set of known human interaction partners and their respective attributes were split into three equal, non-overlapping subsets. Two sets were used to calculate the new maximum points of the information gain for each attribute, and the elements of the remaining third set were used as a test set to evaluate the resulting filters. The number of correctly categorized examples were calculated by summing over all three possible test set.

### Evolutionary analysis

In order to compile evolutionary data on each protein having any putative motif according to our pipeline, we generated a dataset of orthologous sequences. These hits were obtained by running the GOPHER prediction algorithm with default settings against the QFO database [79,80]. Then, we constructed the multiple sequence alignments of orthologs for each protein using the MAFFT algorithm (default parameters) [81]. The ortholog sequences were classified into the most specific term using the five main evolutionary levels according to the UniProt taxonomic lineage: Mammalia, Vertebrata, Metazoa, Fungi and Eukarya.

According to the multiple sequence alignments of orthologs, each aligned instance of the candidate LC8 binding motifs was analyzed in PSSM based conservation terms. For the evolutionary analysis at least two predicted orthologs were required at each level. An aligned motif was considered to be conserved if its PSSM score exceeded the cutoff value (3.3). In the evolutionary filtering step a candidate motif was kept if it was conserved in at least 80% of the mammalian species.

Motif conservation was also analyzed using the SLiMPrints method [19]. This method searches for regions with island-like conservation, i.e. regions that have high conservations relative to their flanking regions. A motif was considered to have an island-like conservation if it had at least 3 overlapping positions with a significant region (p-value<0.05) detected by SLiMPrints. The method was applied to known human binding motifs of LC8 as well as predicted motifs.

In order to study the evolutionary history of the structural organization of AMOT and WWC families, the domain annotation and coiled coil region predictions were retrieved from the InterPro resource (version 63) [82]. The inter-species motif mapping of the known LC8 binding motifs of WWC and AMOT family members was carried out by using the canonical ELM definition and literary data. The definitions of the C-terminal class 3 PDZ-binding motif and the WW-binding motif of group I were obtained from the ELM database [5]. The first WW-binding motif of the AMOT family members was defined as LPxY [74]. In addition, within the orthologues of AMOTL2 the WW-binding motif of group I was defined as PPxF. The identified ortholog sequences were used to generate the multiple sequence alignment and phylogenetic tree of the families by applying the PhyML algorithm (default settings) [83].

### Protein-protein interaction data

To collect PPIs for LC8 from public databases we applied the PSICQUIC approach [43]. Known non-linear motif binding partners (DYNLL1, DYNLL2 and UBC) were omitted. To remove redundant hits, the following four filtering steps were applied: 1. For each partner and study only the direct interactions were kept, except when the given study had only non-direct interactions for that protein. 2. The less informative ‘other affinity chromatography technology’ annotations were filtered out when the study had ‘TAP’, ‘CO-IP’ or ‘pulldown’ annotation as well. 3. Annotations with unknown association type were filtered out if another database had the same interaction from the same study with full annotation. 4. Cases where the PubMed identifier could not be retrieved were filtered out if the given partner was annotated with the same detection method in an identifiable study. The process resulted in a collection of 559 interaction partners for LC8. The partners came from 7 eukaryotic species and 23 virus strains. Finally, for subsequent analysis, the subset of human, mouse and rat data were merged, and this way 381 partners remained. The resulting interaction data is given in S2 Table.

PSICQUIC gives the PSI-MI identifier for detection methods and association types. In the case of the detection methods, based on their meaning and position in the PSI-MI hierarchy, we (i) assigned the labels “biochemical or biophysical”, “PCA” and “other” to them; (ii) merged the detection method descriptions into 8 groups (see the legend on S5A Fig). Similarly, we labelled the association types as “direct” or “non-direct” interaction. PSICQUIC doesn’t provide direct information about the scale of the study, therefore we queried each PubMed identifier in our set. Since the output was, again, redundant, we sorted the number of annotations by source, and took only the highest one. If this number was higher than 50, we considered it as “high throughput”, otherwise as “low throughput”.

### Experimental procedure

The full-length His_6_-tagged LC8 (DYNLL2; UniProt accession number Q96FJ2) was cloned into a pET21-derived pBH4 vector and expressed as described previously [84]. The 11-residue-long fragments of the predicted binding partners were synthesized using solid-phase technique by GenoSphere Ltd.

SPR measurements were performed on a ProteOn XPR36 (Bio-Rad) instrument equipped with HTG sensor chip (Bio-Rad). The sensor chip was activated for 300 s with 150 μl 10 mM NiCl_2_ solution followed by a washing step for 300 s with the running buffer containing 20 mM Hepes, 150 mM NaCl, 0.05% Tween-20, 0.1 mM TCEP, 50 μM EDTA, pH 7.5 buffer. The immobilization of His_6_-tagged LC8 was performed on the activated Ni^2+^-NTA surface at three different densities (1200 RU, 900 RU, 600 RU). The putative binding peptides were injected onto the chip at five different concentrations simultaneously at a flow rate of 60 μl ∙ min^-1^ for 400 s, while the dissociation of the peptides was recorded for 600 s. In the sixth analyte channel, running buffer was injected for double referencing. The double referenced data were global fitted to the 1: 1 Langmuir model using the ProteOn software. The presented K_d_ values were obtained from the mean of the kinetic and the equilibrium analysis delivered K_d_ values, and the standard deviations were calculated from three individual K_d_ values.

## Supporting information

S1 Text Supporting information. (DOCX)

S1 Fig Example structures of the complexes formed between LC8 and selected binding peptides. (DOCX)

S2 Fig Distribution of PSSM scores across the human proteome. (DOCX)

S3 Fig Summarized evolutionary conservation results of known non-human LC8 binding partners. (DOCX)

S4 Fig Summarized evolutionary conservation results of known human LC8 binding motif applying lower PSSM cutoffs. (DOCX)

S5 Fig Statistics of PSICQUIC results. (DOCX)

S1 Table Collection of experimentally verified LC8 binding motifs from the literature. (XLSX)

S2 Table LC8 binding partners from PPI databases. (XLSX)

S3 Table High confidence prediction set for LC8 binding motifs. (XLSX)

S4 Table Experimentally validated LC8 binding motifs. (XLSX)
