# Supplementary material for: Novel linear motif filtering protocol reveals the role of the LC8 dynein light chain in the Hippo pathway
Source: PLoS Comput Biol. 2017 Dec 14;13(12):e1005885. doi: 10.1371/journal.pcbi.1005885 (PMC5746249; doi:10.1371/journal.pcbi.1005885)
Supplement: S1 Text — (DOCX) [file pcbi.1005885.s001.docx]

## **SUPPORTING INFORMATION**

**Database of known interaction partners of LC8**

We assembled a manually curated dataset of LC8 binding motifs based on literature data. A good starting point was a relatively recent review [(1)](https://paperpile.com/c/Er0z4I/jv2SX). Structural complexes formed between known motifs and LC8 were also collected from the Protein Data Bank [(2)](https://paperpile.com/c/Er0z4I/avnY5). Altogether 77 binding motifs had been identified in 61 proteins from various sources. We only considered examples where the interaction was verified both at the protein and the motif level. This criterion lead to the rejection of 10 motifs from 8 proteins due to lack of motif level evidences (S1 Table). Structures of LC8 in complex with various ligands indicate that partners of the domain bind in a relatively similar fashion, including PAK1 which has a different TSP motif instead of the canonical TQT containing motif (S1 Fig). Based on the contacts formed between the binding domain and its ligand as observed in the known structures of complexes, only an 8-residue-long segment is directly involved in the binding.

The dataset of known motifs covered multiple eukaryotic species and viruses. However, the majority of the manually collected binding partners were from human, with additional partners identified in other mammalian species (rat and mouse), or in a cross-species setup. The human and murine homologs of LC8 protein DYNLL1 are identical and the human orthologs of partners identified in other mammalian species could be easily identified due to their high level of conservation even at the level of motifs. In fact, for the seven proteins with motifs identified in murine species (6 from rat and 1 from mouse), the mapped motifs in human were identical, with the exception of the motif in NOS1 protein, which differs in a single position between the human and rat sequences. Hence, we created a human-centric database from the manually curated partners for subsequent analyses that included interaction partners mapped from mouse and rat species. This motif partners (MP) dataset contained verified motif binding sites covering 40 motifs in 33 proteins.


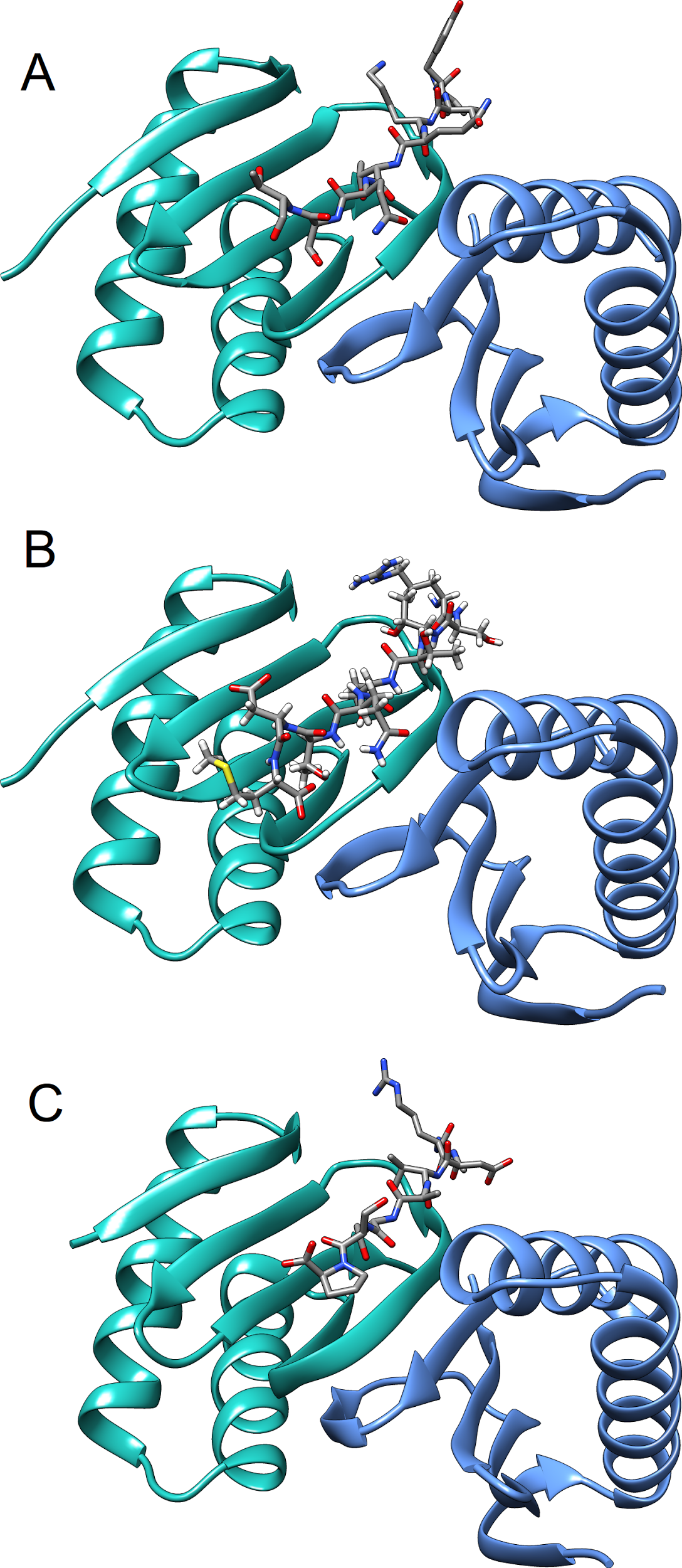


**S1 Fig. Example structures of the complexes formed between LC8 and selected binding peptides.**

(A) LC8 - Intermediate chain complex (PDBID: 2P2T) (B) LC8 - FAM83D complex (PDBID: 5E0M) (C) LC8 - PAK1 complex (PDBID: 3DVT). In all cases, the two LC8 monomers are shown as ribbons and colored light green and light blue, the binding peptides are shown as sticks and colored by elements using the default coloring method. The representation was made with Chimera [(3)](https://paperpile.com/c/Er0z4I/MNcJz).

1. **Distribution of PSSM scores across the human proteome**

Using the obtained PSSM, the whole human proteome was scanned for possible binding motifs. For this, a score was calculated for every overlapping eight amino acid long peptide segment by summing the scores of individual positions. The distribution of the scores for the whole human proteome and the MP set is shown in S2 Fig. By definition, scores above 0 represent cases that are more likely to correspond to true binding motifs. In accordance, all MP motifs had positive scores. While only 2% of human peptides had positive scores, these are expected to be dominated by false positive hits.


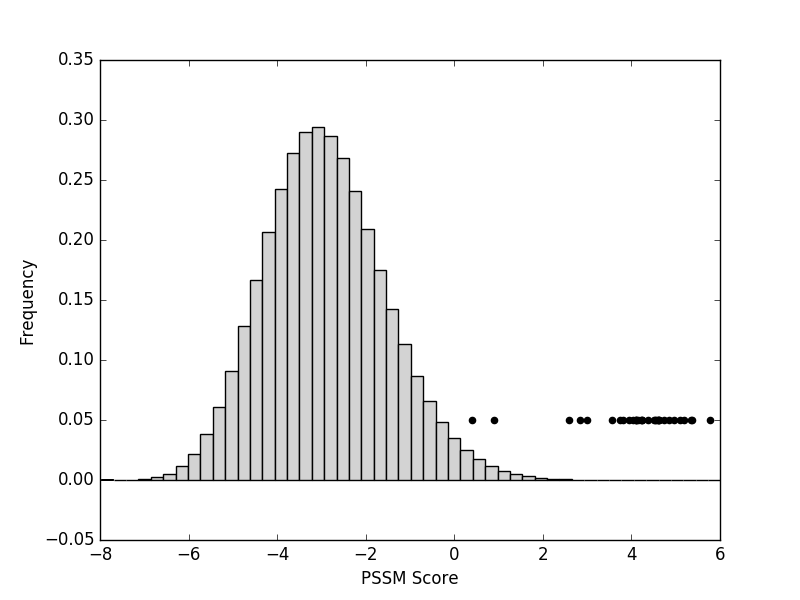
**S2 Fig**. **Distribution of PSSM scores across the human proteome.**
The distribution of PSSM scores calculated for 8 residue long overlapping peptide segments in the human proteome is shown as a histogram. The PSSM scores of the 40 motifs in the MP set are represented as dots.

1. **Evolutionary analysis**


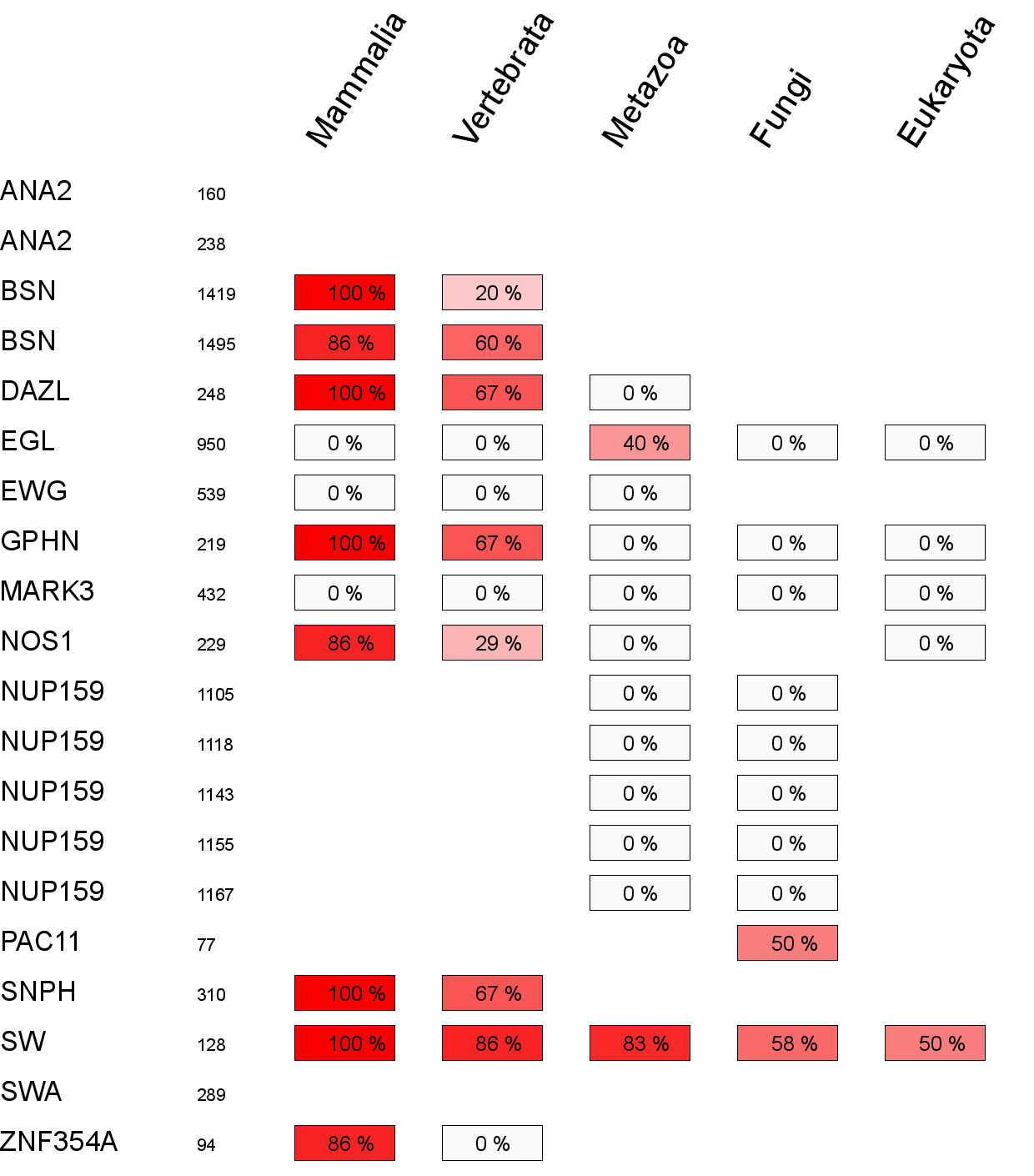


**S3 Fig. Summarized evolutionary conservation results of known non-human LC8 binding partners.** Protein names and motif start positions are indicated in the first and second columns, respectively. The colored boxes represent the presence of orthologues for the known partner at different evolutionary levels. The percentages and colour scheme of the boxes show the PSSM based motif conservation across all species. Conservation values increase from white (low motif conservation) to red (high motif conservation). The partner proteins are derived from four species: Drosophila melanogaster (ANA2, EGL, EWG, SW, SWA), Rattus norvegicus (BSN, GPHN, MARK3, NOS1, SNPH, ZNF354A), Mus musculus (DAZL), Saccharomyces cerevisiae (NUP159, PAC11). No orthologues were predicted for ANA2 and SWA.

**
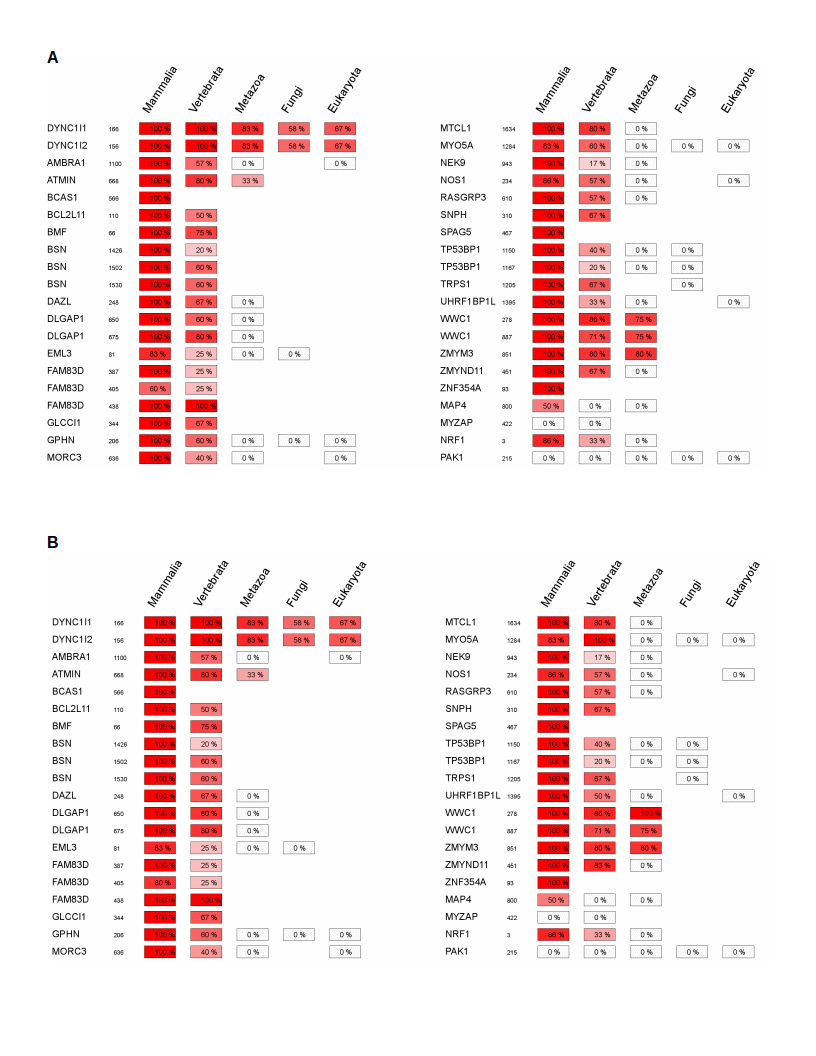
**

**S4 Fig. Summarized evolutionary conservation results of known human LC8 binding motif applying lower PSSM cutoffs.**

Protein names and motif start positions are indicated in the first and second columns, respectively. The colored boxes represent the presence of orthologues for the known partner at different evolutionary levels. The percentages and colour scheme of the boxes show the PSSM based motif conservation across all species. Conservation values increase from white (low motif conservation) to red (high motif conservation). (A) Motif conservation with 3.0 cutoff value. The conservation of NRF1 motifs could be detected with 3.0 cutoff value. In this case the motif of NRF1 was conserved in 86% of mammalian species. (B) Motif conservation with 2.7 cutoff value. At this cutoff value, the second binding motif site of FAM83D motif (at position 405) was detected in 80% of mammals (as opposed to 60% detected with the 3.3 cutoff value) and was detected as conserved. In addition, in vertebrates the conservation of the MYO5A motif also increased to 100%.

1. **Analysis of PPI data**

In order to get an insight into the current knowledge about LC8 interactions regardless of the motif level evidence, we collected interaction partners using the PSICQUIC approach which searches several databases simultaneously (see Materials and Methods). From the found partners, a human-centric database was created by combining mammalian level interactions. Altogether, we collected 381 interaction partners for LC8, supported by 782 experiments altogether.

We compared the interaction data of manually and computationally collected partners in order to gain insights into the basic characteristics of the experimental setup for LC8 partners with known motifs. Known motif partners (MPs) were well represented in current PPI databases, with only three missing out of 33 MP cases. There were 144 experiments corresponding to the known partners (S2 Table). The most common methods used to find LC8 partners were tandem affinity purification, yeast two hybrid methods and co-immunoprecipitation. Interestingly, there were no major differences in the distribution of methods between the interaction partner (IP) set and the motif partner (MP) subset (S5A Fig). The IP dataset was dominated by single experiments, while partners from the MP set were usually verified by multiple methods, with 20 partners identified by at least three methods (S5B Fig). The largest number of experiments (16) supported the interaction of LC8 with the proapoptotic protein Bim (BCL2L11). When we assigned simplified labels to each partner regarding their detection methods, partners with known motifs were more frequently studied by complementary techniques (S5C Fig). Regarding the type of the interaction, physical interaction and association were most common. These terms mean that the interacting pair co-occurs in a larger complex, but there is no proof for direct binary interaction. In the case of the MP set, however, almost half of the partners were detected by direct interaction (S5D Fig). While most of the PPI partners were identified only by high-throughput methods, more than half of the LC8 partners with known motifs were identified by low-throughput methods (S5E Fig). The case of LC8 suggests that there is no single criterion that pinpoints biologically relevant interactions, although PPIs from low-throughput and direct experiments are more likely to be biologically relevant, especially when there are multiple independent measurements.


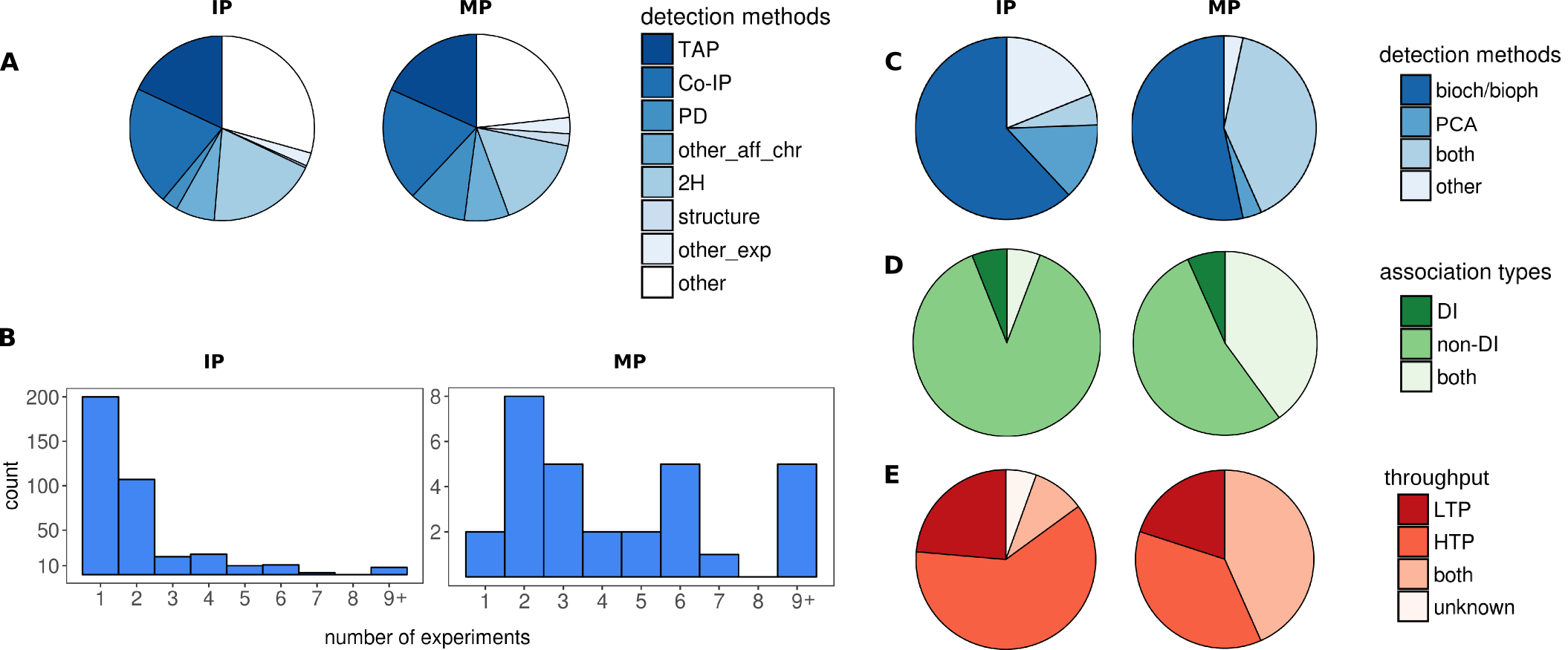


**S5 Fig. Statistics of PSICQUIC results.**

(A) Distribution of detection methods. Partners can occur in more than one category, so the total numbers of experiments are 782 and 144 in the IP and MP sets, respectively. (B) Distribution of the number of partners by the number of experiments they are involved in. Number of partners are shown by features as detection methods (C), association types (D) and studies (E), where each partner appears only once. The total number of partners is 381 and 30 in the IP and MP sets, respectively. IP: interaction partners; MP: motif partners. TAP: tandem affinity purification; Co-IP: co-immunoprecipitation; PD: pulldown; other_aff_chr: other affinity chromatography method; 2H: two hybrid; other_exp: other experimental method. PCA: protein complementation assay. DI: direct interaction. LTP: low-throughput; HTP: high-throughput.

1. **Experimental verification of selected peptide-LC8 interactions**

From the set of candidate motifs, six peptides were selected for experimental validation. Three of the selected peptides (ZMYM4, CIZ1 and IFFO1) were also part of the high confidence list, supported by PPI data or island-like conservation. The selected peptides were chemically synthesized and their binding to LC8 was characterized by SPR assay. The list of selected peptides is shown in S4 Table together with the measured binding constants that confirm the binding of the selected peptides to LC8. These novel examples also increased the coverage of the sequence space of LC8 binding motifs by introducing a novel amino acid, which was previously unobserved among the LC8 binding motifs. For example, the putative binding motif in CCSER2 introduced a Tyr at position -2, MPHOSPH9 had a Trp at position -3, and the motif of CIZ1 had a Pro at position -4.

**S4 Table. Experimentally validated LC8 binding motifs.**

| **Gene name** | **UniProt Accession** | **Start** | **Binding motif sequence** | **K_d_(𝛍M)±SD** |
| --- | --- | --- | --- | --- |
| ZMYM4 | Q5VZL5 | 921 | GDASTQTD | 5.91±1.20 |
| CIZ1 | Q9ULV3 | 289 | VPKQTQTP | 3.93±0.69 |
| IFFO1 | Q0D2I5 | 122 | RDQAVQTG | 0.97±0.17 |
| CCSER2 | Q9H7U1 | 763 | ADKYTQTP | 0.06±0.01 |
| MPHOSPH9 | Q99550 | 909 | KNWGTQTE | 1.20±0.19 |
| CCDC66 | A2RUB6 | 608 | KDTGVQTD | 0.45±0.05 |

**References**

1. [Rapali P, Szenes Á, Radnai L, Bakos A, Pál G, Nyitray L. DYNLL/LC8: a light chain subunit of the dynein motor complex and beyond. FEBS J. 2011 Sep;278(17):2980–96.](http://paperpile.com/b/Er0z4I/jv2SX)

2. [Berman HM, Westbrook J, Feng Z, Gilliland G, Bhat TN, Weissig H, et al. The Protein Data Bank. Nucleic Acids Res. 2000 Jan 1;28(1):235–42.](http://paperpile.com/b/Er0z4I/avnY5)

3. [Pettersen EF, Goddard TD, Huang CC, Couch GS, Greenblatt DM, Meng EC, et al. UCSF Chimera--a visualization system for exploratory research and analysis. J Comput Chem. 2004 Oct;25(13):1605–12.](http://paperpile.com/b/Er0z4I/MNcJz)
