# Supplementary material for: Novel linear motif filtering protocol reveals the role of the LC8 dynein light chain in the Hippo pathway
Source: PLoS Comput Biol. 2017 Dec 14;13(12):e1005885. doi: 10.1371/journal.pcbi.1005885 (PMC5746249; doi:10.1371/journal.pcbi.1005885)
Supplement: S1 Fig — (DOCX) [file pcbi.1005885.s002.docx]

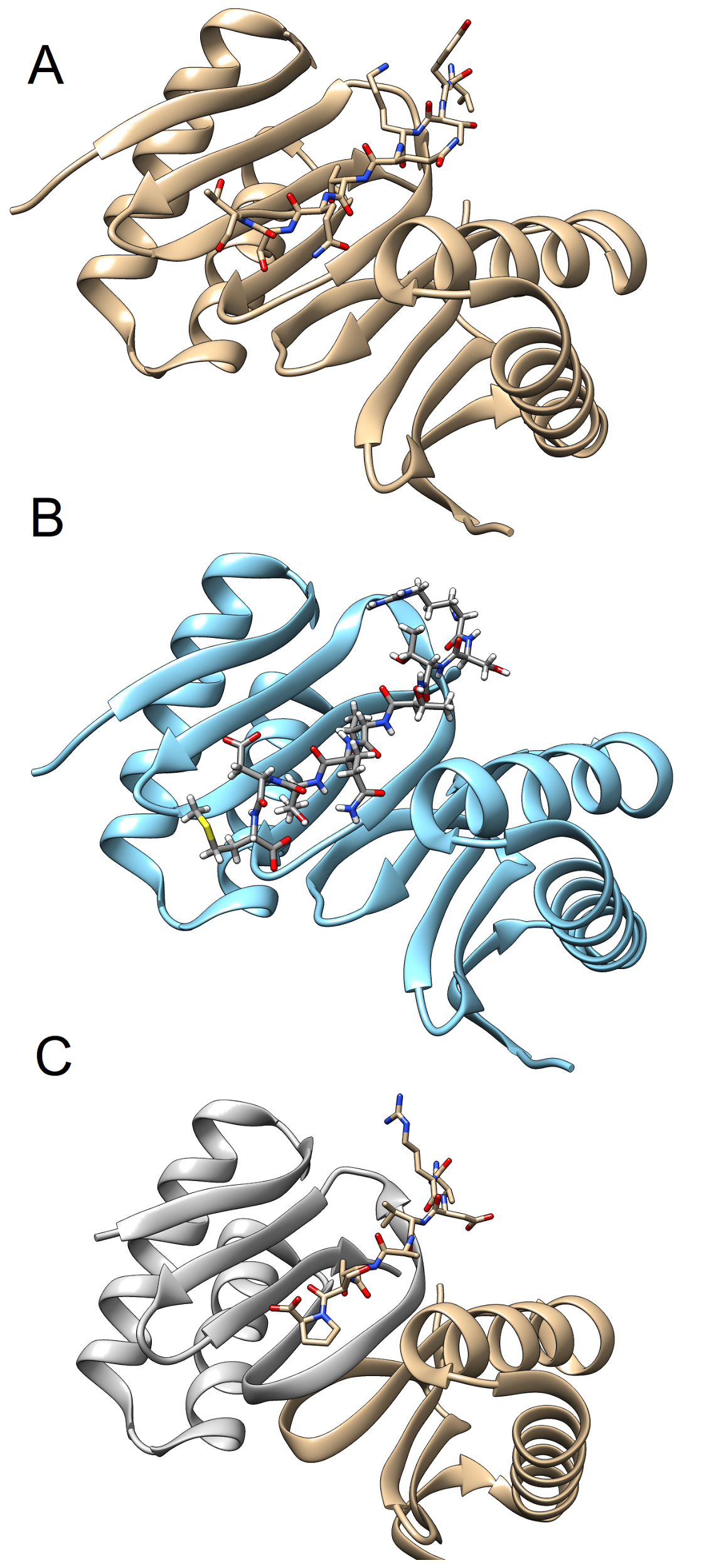


**S1 Fig. LC8 complex structures**

(A) LC8 - Intermediate chain complex (PDBID: 2P2T) (B) LC8 - FAM83D complex (PDBID: 5E0M) (C) LC8 - PAK1 complex (PDBID: 3DVT)
