# Supplementary material for: Novel linear motif filtering protocol reveals the role of the LC8 dynein light chain in the Hippo pathway
Source: PLoS Comput Biol. 2017 Dec 14;13(12):e1005885. doi: 10.1371/journal.pcbi.1005885 (PMC5746249; doi:10.1371/journal.pcbi.1005885)
Supplement: S2 Fig — (DOCX) [file pcbi.1005885.s003.docx]

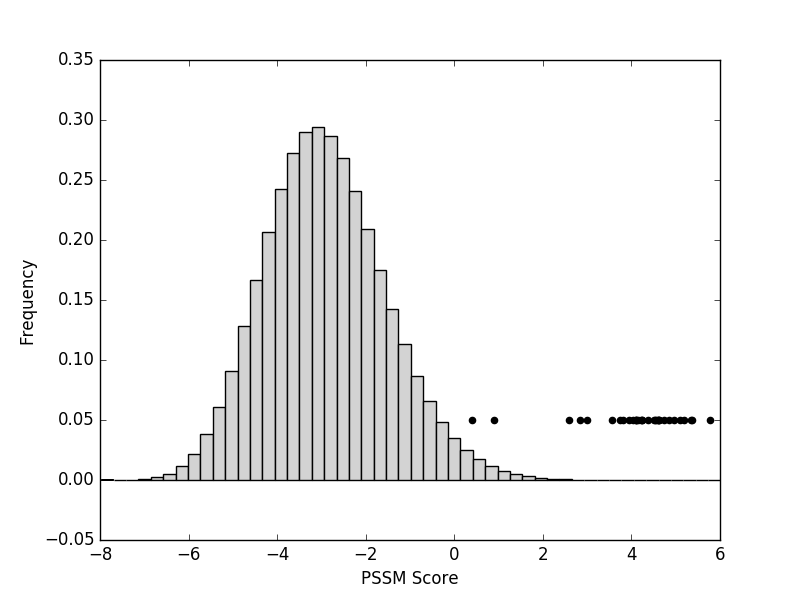


**S2 Fig**. **Distribution of PSSM scores across the human proteome.**
The distribution of 8 residue long overlapping peptide segments in the human proteome scored with the PSSM is shown as a histogram. The PSSM scores of the 40 motifs in the MP set are represented as dots.
