# Supplementary material for: Novel linear motif filtering protocol reveals the role of the LC8 dynein light chain in the Hippo pathway
Source: PLoS Comput Biol. 2017 Dec 14;13(12):e1005885. doi: 10.1371/journal.pcbi.1005885 (PMC5746249; doi:10.1371/journal.pcbi.1005885)
Supplement: S3 Fig — (DOCX) [file pcbi.1005885.s004.docx]

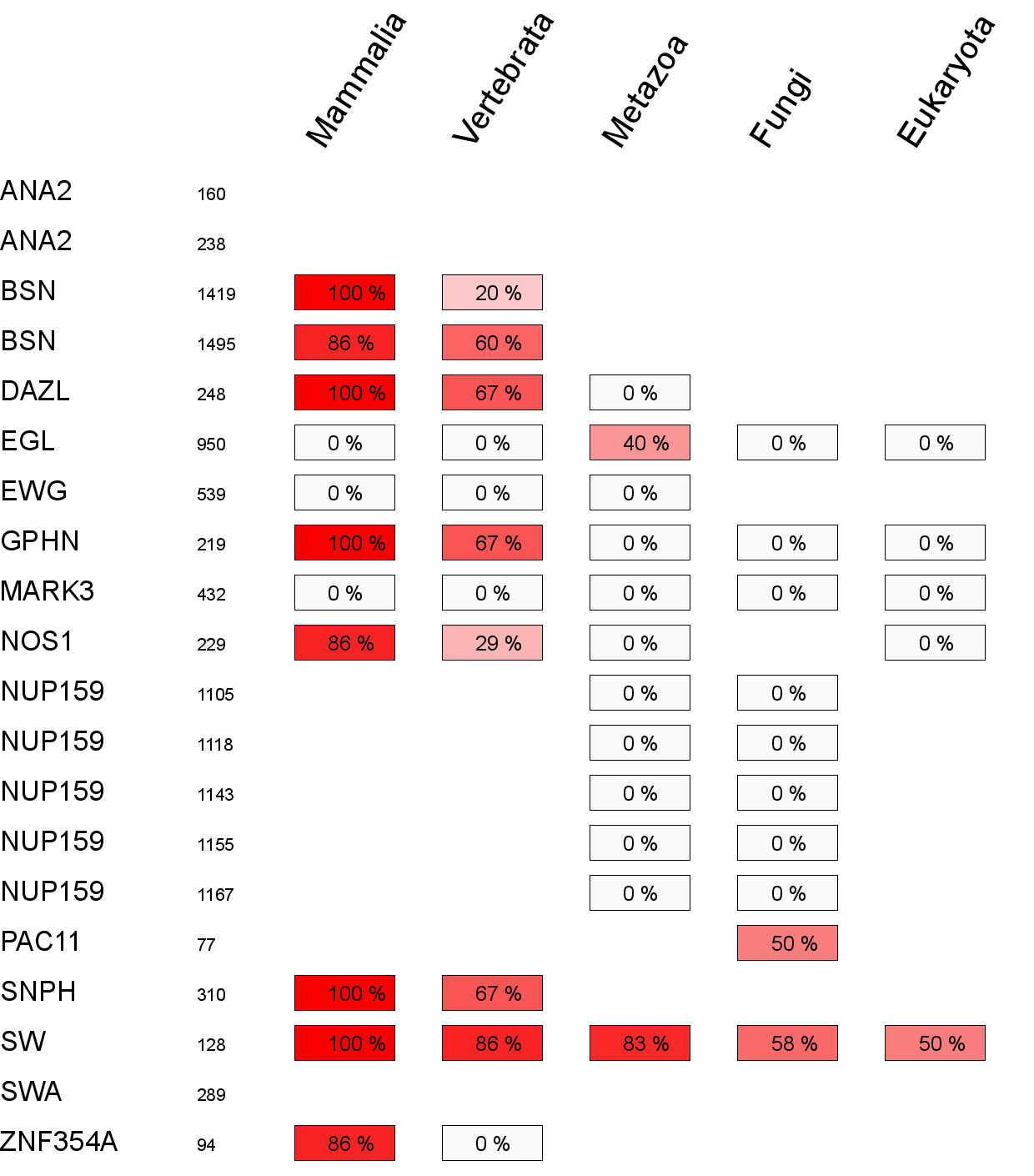


**S3 Fig. Summarized evolutionary conservation results of known non-human LC8 binding partners.** Protein names and motif start positions are indicated in the first and second columns, respectively. The colored boxes represent the presence of orthologues for the known partner at different evolutionary levels. The percentages and colour scheme of the boxes show the PSSM based motif conservation across all species. Conservation values increase from white (low motif conservation) to red (high motif conservation). The partner proteins are derived from four species: Drosophila melanogaster (ANA2, EGL, EWG, SW, SWA), Rattus norvegicus (BSN, GPHN, MARK3, NOS1, SNPH, ZNF354A), Mus musculus (DAZL), Saccharomyces cerevisiae (NUP159, PAC11). Any orthologues of ANA2 and SWA were not predicted.
