# Supplementary material for: Novel linear motif filtering protocol reveals the role of the LC8 dynein light chain in the Hippo pathway
Source: PLoS Comput Biol. 2017 Dec 14;13(12):e1005885. doi: 10.1371/journal.pcbi.1005885 (PMC5746249; doi:10.1371/journal.pcbi.1005885)
Supplement: S4 Fig — (DOCX) [file pcbi.1005885.s005.docx]

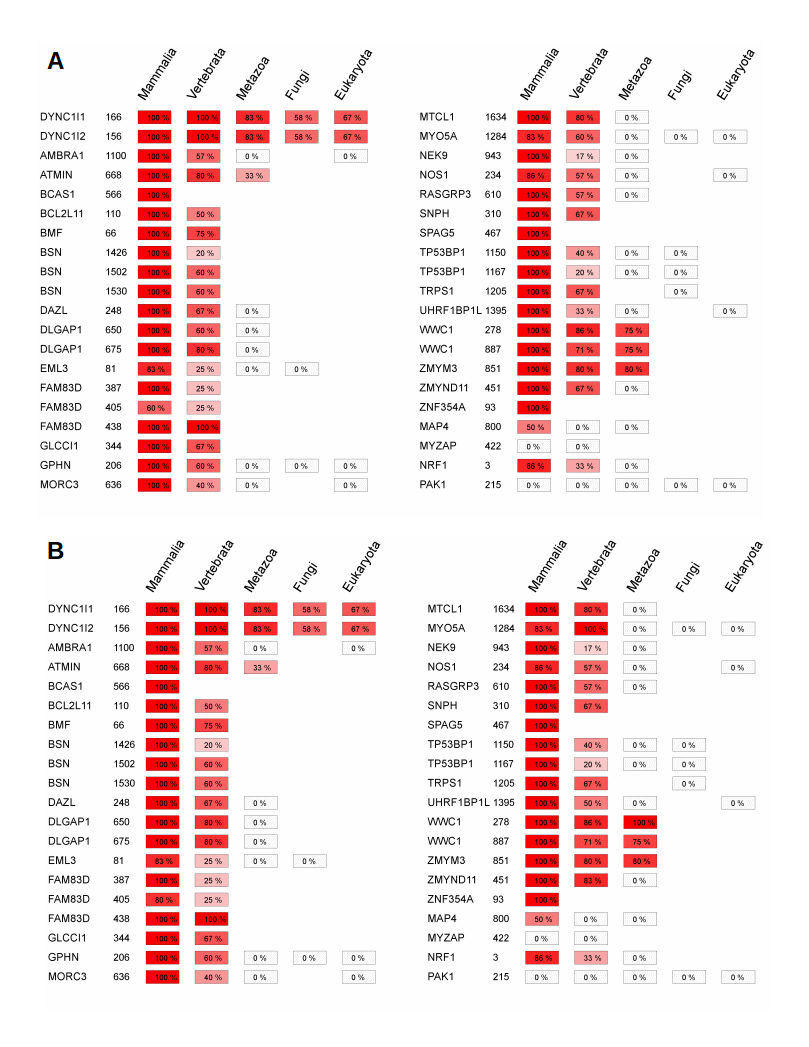


**S4 Fig. Summarized evolutionary conservation results of known human LC8 binding motif applying lower PSSM cutoffs.**

Protein names and motif start positions are indicated in the first and second columns, respectively. The colored boxes represent the presence of orthologues for the known partner at different evolutionary levels. The percentages and colour scheme of the boxes show the PSSM based motif conservation across all species. Conservation values increase from white (low motif conservation) to red (high motif conservation). (A) Motif conservation with 3.0 cutoff value. The conservation of NRF1 motifs could be detected with 3.0 cutoff value. In this case the motif of NRF1 was conserved in 86% of mammalian species. (B) Motif conservation with 2.7 cutoff value. At this cutoff value, the second binding motif site of FAM83D motif (at position 405) was detected in 80% of mammals (as opposed to 60% detected with the 3.3 cutoff value) and was detected as conserved. In addition, in vertebrates the conservation of the MYO5A motif also increased to 100%.
