# Supplementary material for: Novel linear motif filtering protocol reveals the role of the LC8 dynein light chain in the Hippo pathway
Source: PLoS Comput Biol. 2017 Dec 14;13(12):e1005885. doi: 10.1371/journal.pcbi.1005885 (PMC5746249; doi:10.1371/journal.pcbi.1005885)
Supplement: S5 Fig — (DOCX) [file pcbi.1005885.s006.docx]

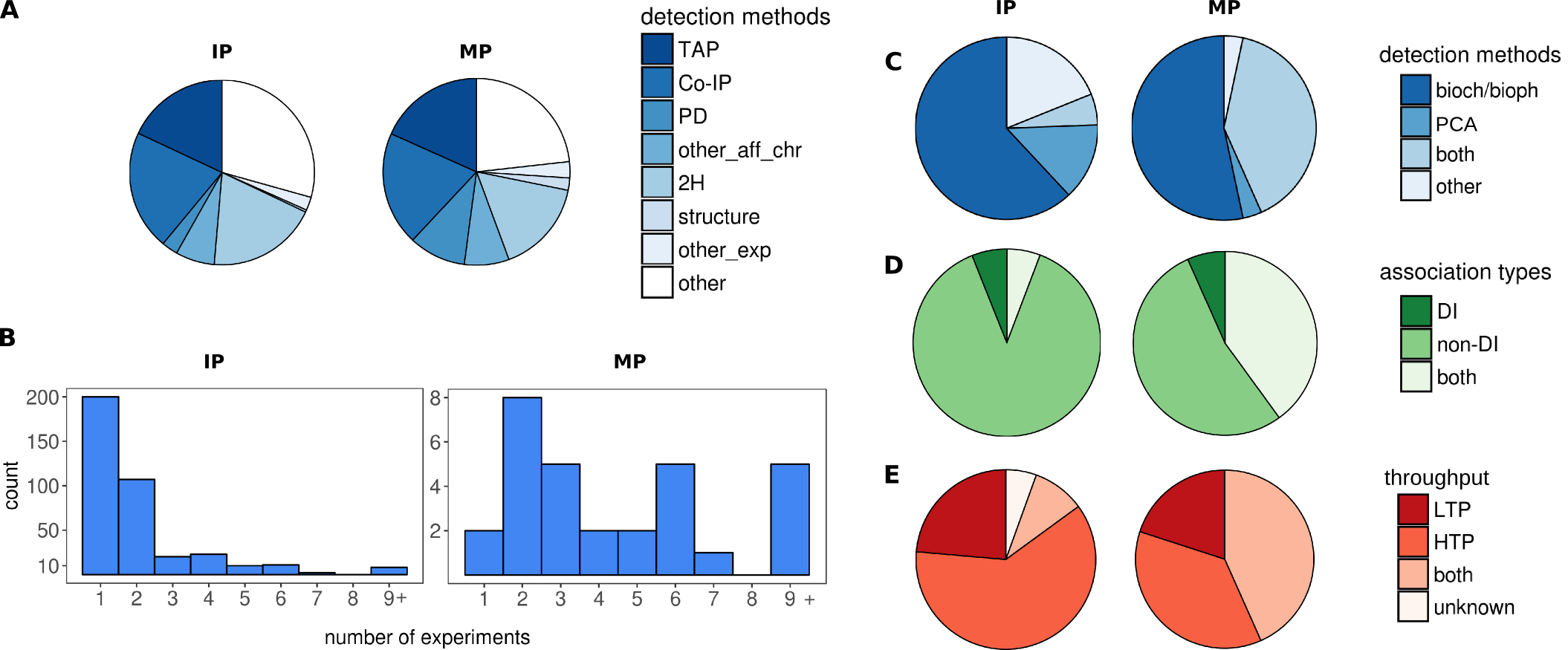


**S5 Fig. Statistics of PSICQUIC results.**

(A) Distribution of detection methods. Partners can occur in more than one categories, so that the total number of experiments is 782 and 144 in the IP and MP sets, respectively. (B) Distribution of the number of partners by the number of experiments they are involved in. Number of partners are shown by features as detection methods (C), association types (D) and studies (E), where each partner appears only once. The total number of partners is 381 and 30 in the IP and MP sets, respectively. IP: interaction partners; MP: motif partners. TAP: tandem affinity purification; Co-IP: co-immunoprecipitation; PD: pulldown; other_aff_chr: other affinity chromatography method; 2H: two hybrid; other_exp: other experimental method. PCA: protein complementation assay. DI: direct interaction. LTP: low-throughput; HTP: high-throughput.
